# Building gene regulatory networks from scATAC-seq and scRNA-seq using Linked Self Organizing Maps

**DOI:** 10.1371/journal.pcbi.1006555

**Published:** 2019-11-04

**Authors:** Camden Jansen, Ricardo N. Ramirez, Nicole C. El-Ali, David Gomez-Cabrero, Jesper Tegner, Matthias Merkenschlager, Ana Conesa, Ali Mortazavi

**Affiliations:** 1 Developmental and Cell Biology, University of California Irvine, Irvine, California, United States of America; 2 Center for Complex Biological Systems, University of California Irvine, Irvine, California, United States of America; 3 Unit of Computational Medicine, Department of Medicine, Solna, Center for Molecular Medicine, Karolinska Institutet, Stockholm, Sweden; 4 Mucosal and Salivary Biology Division, King’s College London Dental Institute, London United Kingdom; 5 Science for Life Laboratory, Solna, Sweden; 6 Biological and Environmental Sciences and Engineering Division, Computer, Electrical and Mathematical Sciences and Engineering Division, King Abdullah University of Science and Technology (KAUST), Thuwal, Kingdom of Saudi Arabia; 7 MRC London Institute of Medical Sciences, Imperial College London, Hammersmith Hospital Campus, London, United Kingdom; 8 Microbiology and Cell Science Department, Institute of Food and Agricultural Sciences, University of Florida, Gainesville, Florida, United States of America; Memorial Sloan-Kettering Cancer Center, UNITED STATES

## Abstract

Rapid advances in single-cell assays have outpaced methods for analysis of those data types. Different single-cell assays show extensive variation in sensitivity and signal to noise levels. In particular, scATAC-seq generates extremely sparse and noisy datasets. Existing methods developed to analyze this data require cells amenable to pseudo-time analysis or require datasets with drastically different cell-types. We describe a novel approach using self-organizing maps (SOM) to link scATAC-seq regions with scRNA-seq genes that overcomes these challenges and can generate draft regulatory networks. Our SOMatic package generates chromatin and gene expression SOMs separately and combines them using a linking function. We applied SOMatic on a mouse pre-B cell differentiation time-course using controlled Ikaros over-expression to recover gene ontology enrichments, identify motifs in genomic regions showing similar single-cell profiles, and generate a gene regulatory network that both recovers known interactions and predicts new Ikaros targets during the differentiation process. The ability of linked SOMs to detect emergent properties from multiple types of highly-dimensional genomic data with very different signal properties opens new avenues for integrative analysis of heterogeneous data.

## Author summary

Gene expression is a tightly controlled process occurring in all cells during all stages of organismal life. How much and when genes are expressed is determined by gene regulatory networks (GRNs), which encode the biological programs that cells can perform. Each cell in an organism is constantly running through these networks to carry out its particular function. New techniques allow us to measure gene expression and chromatin accessibility using single-cell RNA-seq (scRNA-seq) and single-cell ATAC-seq (scATAC-seq). However, these techniques have relatively poor and different signal-to-noise ratios. In this work, we use a form of unsupervised learning called Self-Organizing Maps (SOMs) to analyze one step of B cell differentiation by linking separately trained scRNA-seq and scATAC-seq SOMs. We mine the linked SOMs to reconstruct the underlying GRN using a top-down approach. The resulting GRN not only recapitulated known regulatory linkages but also identified a large number of potential regulatory connections to the system. These methods should be generally applicable to linking heterogeneous high-throughput data with different signal-to-noise profiles.

## Introduction

The ability to analyze hundreds to thousands of individual cells using new functional sequencing assays has revolutionized the current state of scientific and biomedical research [1]. For example, single-cell gene expression studies have allowed the identification of rare cell populations in a variety of samples ranging from immune cell systems [2] to circulating tumor cells [3]. Comprehensive atlases of gene expression are being built for tissues such as the Drosophila brain throughout its lifespan [4] to an entire mouse [5]. Inspired by the wealth of new insights from single-cell RNA-seq, there has been a plethora of single cell genomic technologies developed in the last few years [6]. For example, single-cell profiling of chromatin accessibility [7–9] has generated a lot of excitement because of the wealth of insights generated within large scale surveys of chromatin accessibility and gene regulation through projects like ENCODE [10].

However, unlike single-cell RNA-seq, chromatin accessibility mapping from individual cells yields sparse information of the open chromatin landscape [11,12] due to the intrinsic limitation of numbers of chromosomes per nucleus. It has been difficult for previous analysis platforms to handle the sparsity and noise inherent in data of this type.

Recently, a number of tools have been developed to try and combat this issue. chromVAR^13^ uses cells with the highest proportion of reads to build a model of the expected number of fragments per total reads for every respective motif site in the genome, and computes deviation scores from this model to cluster single cells. This method, while effective, requires the generation of a list of transcription factor binding sites through mass motif scanning which, in this work, necessitated the loosening of strict Type I error control and the creation of a custom, well-curated list of transcription factor motifs. Another application, scABC [13], manages to cluster cells of different cell-types well by using the total cell accessibility signal to provide weights to an unsupervised clustering of the cells using K-medioids and thus identifies landmark regions that are only accessible in each identified population. The cells are then re-clustered using the respective landmarks. However, this technique would likely become confused by time course data from the same cell-type as it may be too similar to generate proper landmarks. BROCKMAN [14] uses gapped 8-mer factorization to calculate variation in DNA sequences in reads across scATAC-seq experiments and can separate cell types across multiple scATAC experiments to determine TF activity through mapping of known TF motifs with gapped k-mers. Unfortunately some TF motifs such as Ikaros (which has only a 5bp motif) are difficult to map properly with a gapped 8-mer. Others, such as Cis-topic [15], were designed to cluster scATAC-seq data alone, but have not been shown to work on multiple data types simultaneously or are only capable of clustering the cells in these experiments, such as latent semantic indexing [16,17].

Additional recent techniques attempt to correct for the scarcity of scATAC-seq data by leveraging imputed pseudo-time orderings [18]. For example, Cicero [19] uses the ordering of cells to make small aggregate pools before computing correlations. Alternatively in a study of human hematopoietic cell differentiation, Buenrostro and colleagues [20] also assigned pseudotime ordering so that accessibility peaks could be smoothed by a lowess function. Both of these methods make extensive use of pseudotime orderings, and thus, require systems that have a strong differentiation lineage (with preferably known markers). Here we introduce a method for jointly analyzing scRNA-seq and scATAC-seq data that cannot be ordered by pseudotime by taking a “gene/region-centric” approach using self-organizing maps.

Self-organizing maps (SOMs) are a type of artificial neural network, also referred to as a Kohonen network [21,22] (S1 Fig). SOMs are trained using unsupervised learning to generate a low-dimensional representation of data and can be visualized using two-dimensional maps. Individual SOM nodes (or neurons) have a weight vector that is in the same dimension as the input data vectors and neighboring nodes on a SOM reflect similarities across the input data space vector. Additionally, SOMs have been known to provide extremely robust clusterings, with typical Rand indexes in the 99.9% range at the unit level and 95% range for continuity-constrained metaclustering [23]. Thus, trained SOMs provide an intuitive platform for identifying clusters in high-dimensional datasets. For example, SOMs trained on gene expression data or chromatin data [24] from multiple cell types in human and mouse have identified complex relationships across high-dimensional genomic data [10,25–27]. Additionally, SOMs have been used to structure and interrogate the transcriptome in single-cells during cellular reprogramming [28]. SOMs provide a natural visual and powerful platform for the analysis and integration of high-dimensional data of different types.

As part of our work in the STATegra consortium (STATegra.eu), we performed single-cell RNA-seq and single-cell ATAC-seq using an *in vitro* mouse pre-B cell model system [29] during cellular differentiation. This system provides a high-resolution view into a narrow transition in pre-B cell development, whereby we induce cell differentiation in response to a sudden doubling of Ikaros expression. Our data only contains two time points and represents a fairly drastic change in chromatin accessibility and gene expression over that period, and thus, would be a poor candidate for pseudo-time analysis. In addition, this data is sufficiently sparse and noisy to give algorithms like UMAP [30] difficulty from a gene or genome region perspective (S2 and S3 Figs) even if the RNA-seq data can be visualized and clustered from a cell perspective (S4 Fig). In addition, we found that Seurat v3 [31], a powerful co-embedding algorithm, also had difficulty in separating the chromatin states using gene expression cell clusters (S5 Fig).

We used SOMatic to create two SOMs in order to identify significant groups of expressed genes and chromatin elements that jointly change during the time course. The two SOMs were then linked using a novel algorithm to find metaclusters of genes and associated genomic regions that show similar profiles during pre-B cell differentiation. The regulatory regions in these clusters were mined for enriched motifs that allowed us to infer a predicted regulatory network downstream of Ikaros. Our flexible and comprehensive approach is first of its kind to provide an analysis platform that combines these, scRNA-seq and scATAC-seq, single-cell data types without leveraging cell ordering and effectively identifies regulatory programs.

## Results

### Integration of single-cell data types using SOM

**Fig 1:**
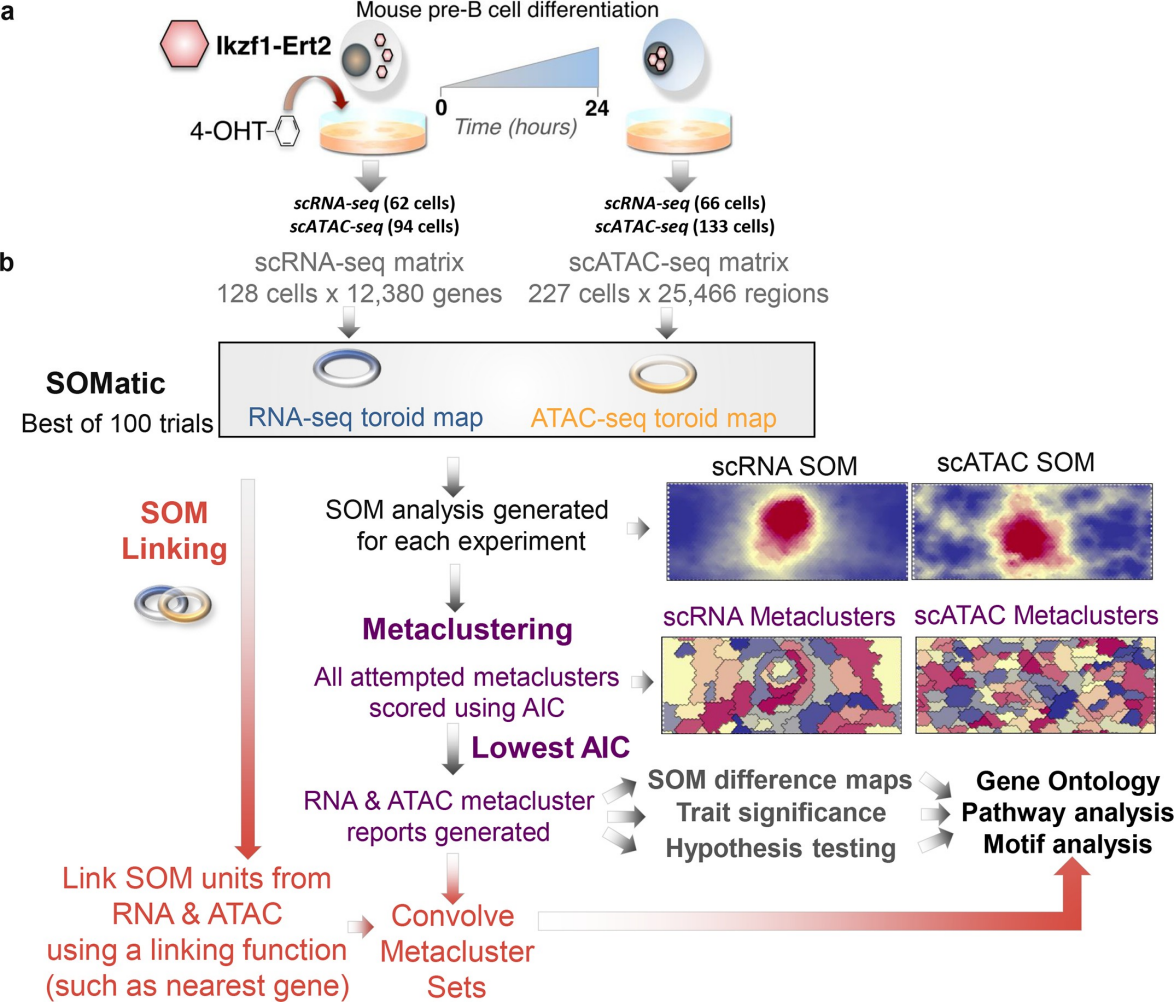
Single–cell multi–data integration using SOMs. (A) An inducible Ikaros mouse pre–B cell–line was used to track changes in gene expression and chromatin accessibility during differentiation (0 and 24–hours) in single–cells. (B) Single–cell RNA–seq and ATAC–seq data from an inducible mouse pre–B cell–line were independently trained using SOMatic to generate single–cell SOMs and metaclustered using AIC scoring. These clusters were convolved with the new SOM linking algorithm to generate pair–wise metaclusters of chromatin regions with similar profiles across the single–cell dataset that regulate genes that also share similar profiles. These pair–wise clusters were mined for regulatory connections through motif enrichment analysis.

In order to study changes in gene expression and chromatin accessibility for single-cells, we utilized an inducible pre-B model system [29] and performed single-cell RNA-seq and single-cell ATAC-seq before and after cellular differentiation (Experimental methods). The goal was to link the data from these methods in a meaningful way to study individual genome region/gene interactions, and this was accomplished by developing the computational pipeline shown in Fig 1. We began by training separate self-organizing maps (SOMs) for each dataset. The result is a set of SOM units that contain genes and genome regions that have a very similar signal profile across each of the single cells at both time points (Summary maps in S6 Fig). To reduce the signal dropout and technical noise prevalent in single cell data, our SOM analysis tool produces clusters of these units, called metaclusters [24], which maintain the SOM’s scaffold topology by only combining adjacent units and contain similar gene expression and chromatin accessibility profiles. Finally we combine the patterns found in each SOM using a pipeline that links metaclusters from both gene expression and chromatin accessibility. These linked metaclusters (LM) contain sets of chromatin regions that have similar open chromatin signal profiles that are in the proximity of genes that also share a similar profile (although not necessarily the same profile in RNA and ATAC) and can be mined using gene ontology, pathway analysis, and motif discovery. Our method easily extends a traditional single data-type analysis to one that focuses on the integration of fundamentally different data like single-cell RNA-seq and ATAC-seq in order to recover evidence of co-regulation.

### Identification of dynamic gene expression metaclusters

**Fig 2:**
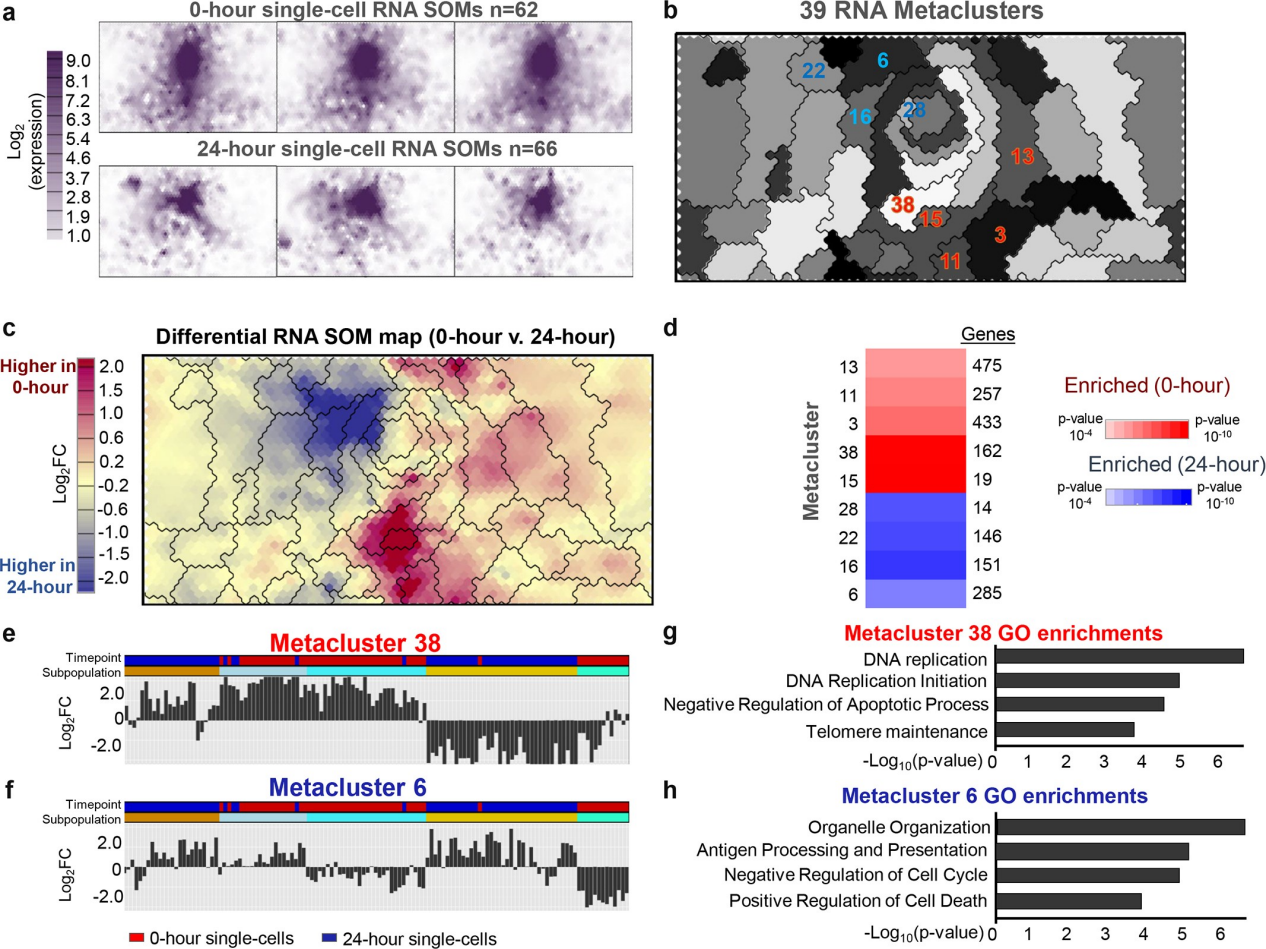
Single–cell gene expression patterns during cellular differentiation are profiled using SOMatic. (A) A SOM was generated for the single–cell RNA–seq dataset (0–hour 62 cells, 24–hour 66 cells). Maps for 3 cells from each time point were arbitrarily selected for display. (B) 39 metaclusters were identified using AIC scoring. Metacluster number and color were arbitrarily assigned for visualization purposes. (C) SOM difference map comparing 0–hour and 24–hour time–points. Maps for cells from 0 and 24–hour timepoints were averaged to generate a single map for each and then subtracted to create a map that represented gene expression fold change during pre–B cell development. Overlaid metacluster divisions generally follow contours of the map. (D) Trait enrichment analysis deployed on gene metaclusters revealed which are enriched in each time point. Metaclusters of interest are highlighted in panel b. (E–F) Summary showing the representative expression profile for metaclusters 38 and 6. Columns are individual cells color–coded for 0 and 24–hour time–points ordered by hierarchical clustering on every metacluster representative gene expression profile. Cell subpopulations are represented by a 40% cut on that clustering. (G–H) Top gene ontology terms for the 162 genes in metacluster 38 and the 151 genes in metacluster 16.

We trained a 40x60 SOM on the scRNA-seq dataset (62 single-cells for 0-hour; 66 single-cells for 24-hour) using 12,380 genes that had expression greater than 1 FPKM in at least 5% of cells (Experimental methods). As expected, slices of this map (Fig 2A), which correspond to single cells, show a general reduction of gene expression over time. SOMatic identified 39 RNA metaclusters that reflect the various gene expression profiles present in the data (Fig 2B). We validated that these metaclusters were properly determined by calculating the UMatrix and density map for this SOM and overlaying the metacluster boundaries on top of these maps (S7 Fig) for visual inspection. The metaclusters followed the breaks in these maps as expected and thus provide a robust representation of the different profiles present in the single-cell data.

One of the strengths of the SOM approach is that we can perform logical operations on the feature maps. We computed a map by averaging maps from the cells in each time point and subtracting them to determine which metaclusters reflect meaningful gene expression differences across time (Fig 2C). We performed a correlation analysis to determine which metaclusters were consistently enriched across the cells in each time-point as previously described [24]. We found statistically-significant differences across time in 9 RNA metaclusters, 5 of which were enriched in 0-hour and 4 in 24-hour (Fig 2D, p-value <10^−4^–10^−10^). Sizes for these metaclusters can be found in Fig 2D. For example, RNA metacluster 15 consists of 11 SOM units and contains 19 genes enriched in 0-hour single-cells such as *Igll1* and *Vpreb1* (Fig 2E). Similarly, metacluster 16 consists of 42 units and contains 151 genes enriched in 24-hour cells such as *Mier1*, which has been shown to control mature B-cell survival in mice [32] (Fig 2E). Gene ontology analysis revealed a series of genes enriched for antigen presentation and negative regulation of cell cyle in 24-hour cells, while DNA replication genes were represented in 0-hour cells (Fig 2F). This is consistent with the transition of gene programs necessary for coordinating pre-B cell differentiation [33].

### Mapping the pre-B single-cell chromatin landscape architecture using SOMs

We performed single-cell ATAC-seq [8] with a total of 227 cells passing our quality controls to explore the change in chromatin accessibility over the differentiation time-course. We recovered on average 53,864 unique chromatin fragments per cell. Using peaks taken from a set of pooled ATAC-seq experiments over three biological replicates with 50,000 cells for each time-point, we quantified the ATAC-seq signal in these peaks for each cell. We built a data matrix from chromatin regions detected in at least 2% of cells (5 cells) for a total 25,466 ATAC-seq peaks due to the sparse nature of single-cell ATAC-seq.

**Fig 3:**
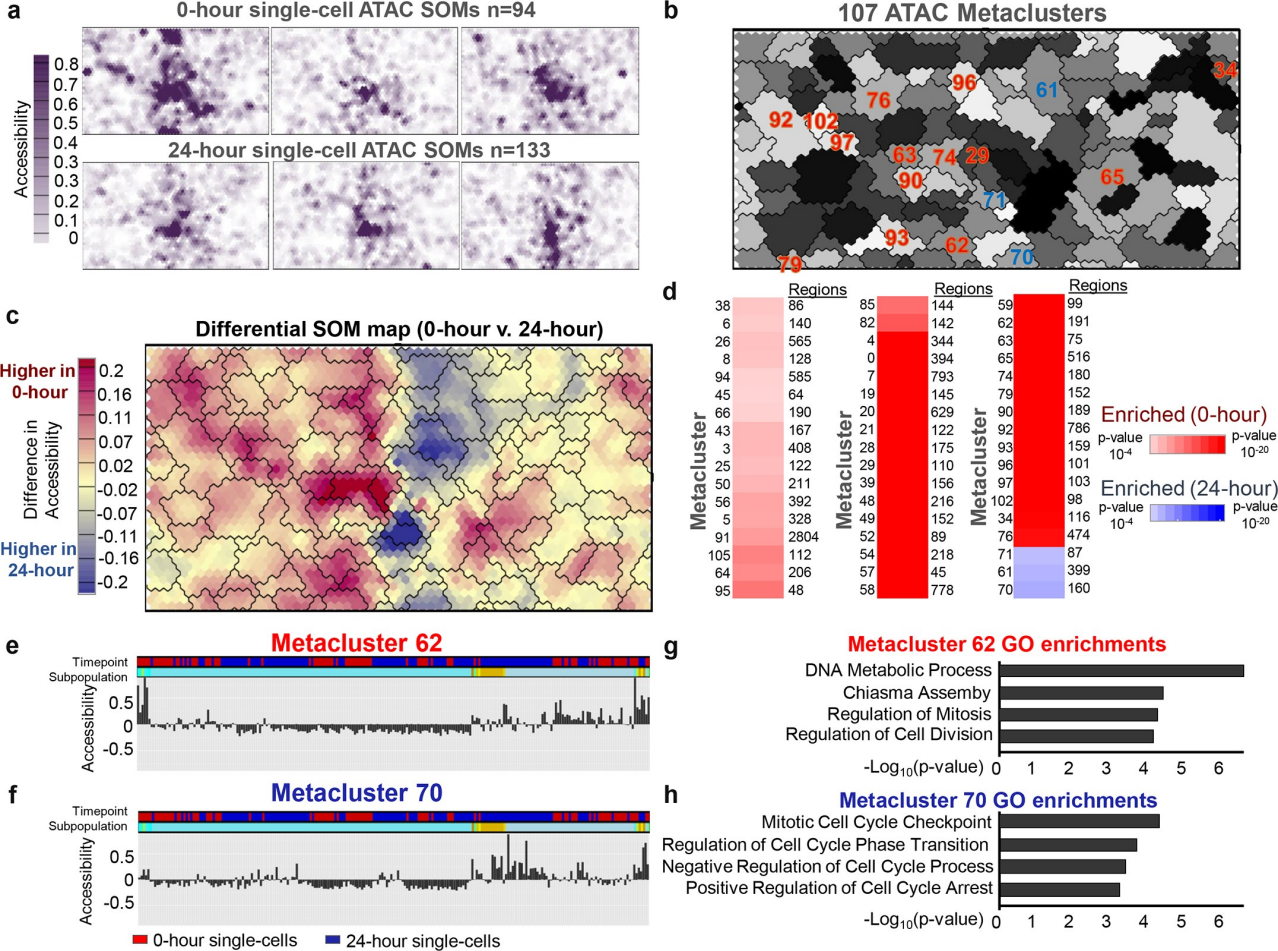
SOMatic reveals the dynamic chromatin landscape in single–cells. (A) A chromatin SOM was generated for the single–cell ATAC–seq dataset (0–hour 94 cells, 24–hour 133 cells). Maps for 3 cells from each timepoint were arbitrarily selected for display. (B) 107 metaclusters were identified using AIC scoring. Metacluster number and color were arbitrarily assigned for visualization purposes. (C) SOM difference map comparing 0–hour and 24–hour time–points. Maps for cells from 0 and 24–hour timepoints were averaged to generate a single map for each and then subtracted to create a map that represented chromatin accessibility fold change during pre–B cell development. Overlaid metacluster divisions generally follow contours of the map. (D) Trait enrichment analysis deployed on gene metaclusters revealed which are enriched in each time point. Metaclusters of interest are highlighted in panel b. (E–F) Summary showing the representative accessibility profile for SOM metaclusters 62 and 70. Columns are individual cells color–coded for 0 and 24–hour time–points ordered by hierarchical clustering on every metacluster representative gene expression profile. Cell subpopulations are represented by a 40% cut on that clustering. (G–H) Top gene ontology terms for genes associated to chromatin elements from SOM metaclusters 62 and 70. Association was determined through use of the GREAT algorithm (See methods).

A 40x60 SOM was trained on this scATAC data matrix (Experimental methods). Similar to the RNA SOM, scATAC feature maps (Fig 3A) revealed a general closing of the chromatin in 24-hour cells, which is normal for cells undergoing differentiation. Clustering the units from this SOM resulted in the identification of 107 chromatin metaclusters (Fig 3B). Visual inspection of these clusters confirmed that these clusters properly follow the breaks in the UMatrix and density map (S8 Fig).

A SOM difference map and hypothesis analysis for all 107 chromatin metaclusters revealed 48 metaclusters that exhibit open chromatin signal in 0-hour cells and 3 metaclusters in with higher signal in the 24-hour cells (Fig 3C and 3D). Gene ontology enrichments for genes in the vicinity of the regions from two of the most significant metaclusters (Fig 3E), 62 (0-hour enriched; 191 peaks) and 70 (24-hour enriched; 160 peaks), reveal that these genes are enriched for cell cycle and cell division programs as predicted (Fig 3F). Thus, SOMs are capable of revealing patterns of chromatin accessibility from sparse single-cell ATAC-seq data in a dynamic model system.

### Comparison of chromatin SOM results to cisTopic clustering

In order to compare the performance of our SOM clustering on the scATAC-seq data, we also analyzed that dataset using cisTopic [15](Experimental Methods), which determined that there were only 15 region clusters (“factors”, S9A Fig). Umaps built using these 15 factors clustered the cells from this experiment into coherent groups (S9B Fig) with several factors (3, 6, 8, 13) being enriched in one timepoint over the other. However, GREAT analysis of these factors did not reveal any significant GO terms that were biologically relevant, which may be due to the large sizes of these topics (S9C Fig). Additionally, visual inspection of the *Igll1* locus (S9D Fig) showed that the promoters of *Igll1* and *Vpreb1* and one of the nearby enhancers with extremely different scATAC-seq profiles were all assigned to the same topic. A global comparison can be found in S10 Fig. Although cisTopic only clustered 8,266 out of the 25,466 regions, the silhouette coefficient is similar to SOMatic from a metacluster perspective (S11 Fig). Thus, while cisTopic performs well in the separation and clustering of cells from scATAC-seq experiments, it is unable to achieve a high-resolution view of the inner-time-point dynamics at the same granularity as the SOM.

### Application of multi-omic single-cell data integration using Linked SOMs

Cellular differentiation occurs as a consequence of dynamics in expression of networks composed of genes controlled by cis-regulatory elements, which must be open in order to function properly. The linker pipeline within SOMatic attempts to convolve the metaclusters from RNA and chromatin accessibility SOMs in order to interrogate the dynamics of the system. In brief, the pipeline subsets chromatin regions within the same chromatin metacluster into linked metaclusters (LM) using the expression of the gene whose regulatory region (using the same algorithm as GREAT [34]) overlaps the element. Thus, if a set of regions are in a LM, these regions share a similar chromatin accessibility profile and are in the vicinity of genes that also share a similar gene expression profile (See S12 Fig for an overview). This coherence of joint profiles gives a much higher expectation that these regions will be similarly regulated than grouping on accessibility or gene expression alone.

**Fig 4:**
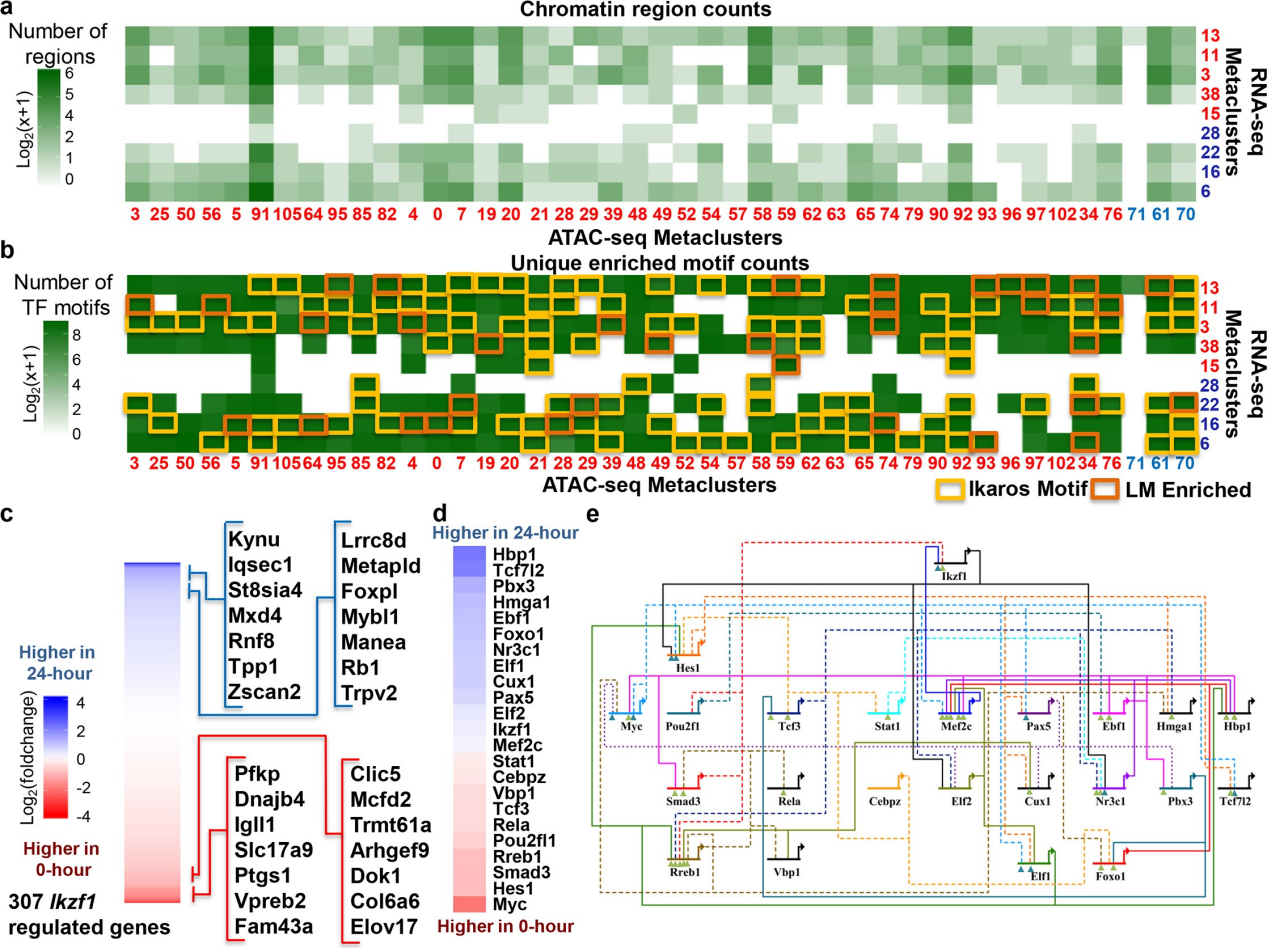
Transcriptional regulation by *Ikzf1* recovered using linked SOMs. (A) Size of pair–wise metaclusters that contain both differentially–expressed genes and differentially–accessible chromatin sites. Metaclusters of genes and regions with a higher enrichment at 24–hours are colored blue and are ordered by enrichment in the two time points. (B) Number of statistically–significant motifs found in each pair–wise metacluster from (a). Presence and enrichment of the *Ikzf1* motif in the pair–wise metacluster is noted. (C) Heatmap of expression fold change for genes predicted to be regulated by *Ikzf1*. Genes with the largest change between time points are noted. (D) Predicted downstream targets of *Ikzf1* with significant change over the time course. Each gene is labels with the fold change between time points with the same scale as 4c. (E) Predicted gene regulatory network downstream of *Ikzf1*. Genes are ordered left to right by their fold change over the time course. Connections are dashed if their signal is significantly lower at the 24–hour time point. Connections at each gene are labeled by level of evidence found in existing literature. Teal triangles indicate experimental evidence and green triangles indicate previous computational prediction.

We applied this new pipeline to our scRNA and scATAC SOMs and analyzed a total of 107 x 39 = 4,173 LMs to identify 459 LMs that were significantly dynamic in both chromatin accessibility and their nearby genes (Experimental methods) (Fig 4A). Based on our assumption that these LMs were similarly regulated, we mined each LM separately for known transcription factor binding site motifs using FIMO with a q-value cutoff of .05. This generated ~9.3 million candidate motifs, which is substantially more than results from standalone motif analysis on bulk data with less than 50k and 500k for peaks and enriched peaks respectively (S13A Fig) and is greater than the ~4.4 million using the ATAC-seq SOM on its own (S13B Fig). Random LMs also gave us fewer candidate motifs, with an average of ~1.46 million motif positions in 100 trials (S14 Fig). Additionally, to determine enrichment, LMs with a percentage of regions containing each transcription factor motif that was significantly (p-value < .05) enriched over the baseline were reported, (Fig 4B), reducing the ~9.3 million candidate motifs to 265,715 high-confidence potential gene regulatory network connections or 5,268 high-confidence active transcription factor/active transcription factor connections.

The differentiation of the B3 cell line is initiated by doubling the amount of Ikaros in the nucleus of each cell and we therefore focused our analysis on Ikaros as the root node of our gene regulatory network. A majority of the differential LMs contain the Ikaros motif (3,672 total instances compared to an average of 1,232 instances in shuffled clusters), including 35 where Ikaros reaches statistical significance (compared to none in the shuffled clusters). In total, we found 307 genes, with 328 nearby potential cis-regulatory regions that contain the motif, that may be regulated directly by Ikaros (Fig 4C), including genes known to be differentially expressed in this system, such as *Igll1* (S15 Fig) and *Vpreb2* [35] as well as the transcription factor *Nr3c1* [36], which is a factor that has been previously implicated as being downstream of Ikaros. To validate these connections, *Ikzf1* ChIP-seq data [33] was interrogated at the same 0hr and 24hr time points for each of the 328 potential cis-regulatory regions. Of these, 312 (~95%) of these regions overlap *Ikzf1* ChIP-seq peaks in one or both of the time points, including 84 (~26%) that overlap *Ikzf1* ChIP-seq peaks in only one time point. Loci for the 3 transcription factors predicted to be regulated by Ikaros were further visually inspected and each of the nearby potential cis-regulatory regions had a significant change over the time course (S16–S18 Figs).

We built a gene regulatory network of transcription factors that we predicted were connected to Ikaros to identify indirect, secondary changes to gene expression as a direct result of changes in Ikaros concentration at the direct targets TFs. This network is tied directly to the model system in that it only uses genome segments that are open in either time-point. We determined which factors downstream of Ikaros showed a significant change in expression across the time-series (Fig 4D) and determined the connections between them (Fig 4E). Each of these genes has been shown to be important in B-cell differentiation. For example, the activation of *Hbp1* has been shown to prevent c-Myc-mediated transcription [37] and, together with a down-regulation of *Myc* expression, stops B-cell proliferation. The temporal enrichment of predicted targets downstream of *Myc* can be found in S19 Fig.

About 16% of connections in this network have been previously described [36,38–41], which include *Mef2c* to Ikaros [42] and *Pax5* and *Myc*’s negative feedback loop [43,44], or have been previously computationally predicted [45–47](52%), and we identify 20 new connections like *Rreb1* to *Myc* (S20 Fig). The identification of both direct and indirect regulation from a sudden doubling of Ikaros demonstrates the power of the Linked SOMs for analyzing highly-dimensional multi-omics data.

### Scalability of the linked SOM for larger datasets

In order to demonstrate the scalability of our approach to larger datasets, we applied the Linked SOM to the recently published sciCar dataset that measured chromatin accessibility and gene expression in the same DEX-treated A549 single cells [48]. This dataset features ~6,000 cells for each experiment with an average of 100,000 reads per cell for the scRNA-seq and 55,000 reads per cell for the scATAC-seq (each of which are an order of magnitude lower than the pre-B cell data above). We built a training matrix of 3,234 cells that passed our filters in both datasets and applied the Linked SOM methodology. (Experimental Methods) We found that the individual RNA and ATAC SOMs called a similar number of DE genes and DA regions to those found in the publication (S12A–S12C Fig). After SOM linking, we measured the correlations on the average signal across each timepoint (0h, 1h, and 3h) in both experiments in each LM and compared the distribution to correlations in the differential LMs and correlations when the timepoints for the cells are randomly scrambled. (S21D Fig). We found that the differential LMs have a lower density of combinations with no correlation and are more skewed towards the positive end, and both distributions are significantly (pvalue < .003) different in the scrambled dataset.

To explore this further, we computed a heatmap of the correlations for the differential LMs (S17E Fig). Investigating the contents of the positively correlated differential LMs revealed the promoter-gene connections for genes known to be targeted by Gluticoid Receptor (GR) activation such as *Ckb*, *Per1*, *Nfkbia*, *Cdh16*, and *Scnn1a*. Additionally, after motif analysis in those LMs, we recovered the motif of Nr3c1 (the GR receptor) in the promoter of each of the above genes (S21F Fig). These results show that Linked SOMs are capable of analyzing data from larger single-cell experiments with fewer reads per cell and can recover biological insights by leveraging separate measurements of RNA expression and chromatin accessibility without leveraging the same cell measurement of sciCar while demonstrating similar results.

We then compared the SOM linking algorithm to the analysis method from the original work using relationships between chromatin accessibility and gene expression present in the sciCar data. First, we performed a pseudotime analysis with monocle [18] on the scRNA-seq data that produced similar results (S22A Fig). Next, we combined the scaled signal in the scATAC-seq and scRNA-seq experiments into 11 evenly-sized batches of pseudotime-ordered cells (290 cells a piece), again with similar results (ZSWIM6 in S22B and S22C Fig). Finally, we calculated the correlations between these binned gene expression profiles and their promoters’ combined chromatin accessibility profile and compared it to the correlations of their linked metacluster profiles (S22D Fig). The linked metaclusters profiles (binned in a similar way) had significantly higher correlations than the scaled gene expression/chromatin accessibility profiles. This suggests that SOM metaclustering does a better job of cleaning up sparse and noisy data than simply scaling and binning cells by pseudotime.

## Discussion

In this work, we used a gene- and chromatin-centric analysis using SOMs on a mouse pre-B time-course data of single-cell RNA-seq and ATAC-seq separately and, then, convolved them to find synergistic effects. Combining the metaclusters from multiple SOMs as a pair-wise set generates a data-space that combines the properties from both without any assumptions about how the data relates to each-other. Due to the inheritance of each SOM’s properties, the linked metaclusters (LMs) contain genome regions that should be similarly regulated: not only is the chromatin accessibility of those regions similar across the cells, but the nearby genes they regulate share expression patterns. Thus, these LMs can be mined for motif enrichment and return a higher number of significant motif sites than simply dividing the data set randomly or by signal changes in either data set separately.

We used this SOM linking technique to explore the regulatory control of the lymphoid regulator *Ikzf1* during one step of B-cell development. 35 LMs enriched in the *Ikzf1* motif contained regions that had similarly-differential chromatin accessibility between time points and had had differentially expressed genes. Our analysis successfully recovers known biology about *Ikzf1* regulation on target genes *Igll1*, *Vpreb2*, and *Nr3c1* and novel regulatory information through discovery of possible downstream mechanisms for B-cell activation. Following the interactions around the network provides many exciting, new avenues for research.

It is important to note, however, that these predicted regulatory connections use an extremely stringent statistical cutoff to be as confident as possible, and thus, do not recover some of the linkages predicted based on *Ikzf1* ChIP data [33] such as Ikaros’s involvement in the regulation of *Myc* and *Foxo1*. While we do detect these connections at an early portion of the pipeline, the genome sequence in those regulatory regions are too different from the canonical motif to pass our stringent filters. *Foxo1* had an Ikaros motif in an open chromatin region near its transcription start site, but the motif only had a q-value of 0.762, which was above the threshold.

Our approach for combining multi-omic data through linked SOMs is amenable to integrating other single-cell technologies for the purpose of multi-omic data analysis as long as a linking function can be found. For example, the profiling of small RNAs, such as miRNAs [49], in single cells could be linked with a standard scRNA-seq experiment through the use of target prediction algorithms. The hypothetical LMs in that case would include groups of miRNAs with similar expression patterns such that their target RNA also has similar expression patterns. Following identification of these groups, functional analysis could be done on each group target RNAs and these functions could be passed back to the miRNA in the group. This is just one example of an exciting experimental and computational design that linked SOMs enable.

The ability to perform multi-omic experiments from a single-cell is now achievable for several biochemical and genomic platforms [50–53] with more being developed every day. We foresee the ability to connect the patterns in multi-omic data using algorithms like linked SOMs to be integral in using this new technology to the fullest.

## Methods

### Pre-B cell differentiation

ERt2-Ikaros inducible B3 cells were cultured in Iscove’s Modified Dulbecco’s Medium (IMDM) supplemented with 10% FBS. Differentiation was induced as previously shown [33]. Briefly, cells were induced with 20mM of 4-hydroxytamoxifen (4OHT), over the course of 24 hours. Prior to performing single-cell experiments, cells were washed twice with cold 1X PBS.

### Single-cell RNA-seq

Single cells were isolated using the Fluidigm C1 System. Single cell C1 runs were completed using the smallest IFC (5–10 um) based on the estimated size of B3 cells. Briefly, cells were collected for 0 (1 batch) and 24-hour (2 batches) time-points at a concentration of 400 cells/μl in a total of 50 μl. To optimize cell capture rates on the C1, buoyancy estimates were optimized prior to each run. Each individual C1 capture site was visually inspected to ensure single-cell capture and cell viability. After visualization, the IFC was loaded with Clontech SMARTer kit lysis, RT, and PCR amplification reagents. After harvesting, cDNA was normalized across all libraries from 0.1–0.3 ng/μl and libraries were constructed using Illumina’s Nextera XT library prep kit per Fluidigm’s protocol. Constructed libraries were multiplexed and purified using AMPure beads. The final multiplexed single-cell library was analyzed on an Agilent 2100 Bioanalyzer for fragment distribution and quantified using Kapa Biosystem’s universal library quantification kit. The library was normalized to 2 nM and sequenced as 75bp paired-end dual indexed reads using Illumina’s NextSeq 500 system at a depth of ~1.0–2.0 million reads per library.

### Single-cell ATAC-seq

Single-cell ATAC-seq was performed using the Fluidigm C1 system as done previously [8]. Briefly, cells were collected for 0 and 24-hours post treatment with tamoxifen, at a concentration of 500 cells/μl in a total of 30–50 μl. Additionally, 3 biological replicates of ~50,000 cells were collected for each measured time-point to generate bulk ATAC-seq measurements. Bulk ATAC-seq was performed as previously described [54]. ATAC-seq peak calling was performed using bulk ATAC-seq samples. ATAC-seq peaks were then used to estimate single-cell ATAC-seq signal. Our C1 single-cell capture efficiency was ~70–80% for our pre-B system. Each individual C1 capture site was visually inspected to ensure single-cell capture. In brief, amplified transposed DNA was collected from all captured single-cells and dual-indexing library preparation was performed. After PCR amplification of single-cell libraries, all subsequent libraries were pooled and purified using a single MinElute PCR purification (Qiagen). The pooled library was run on a Bioanalyzer and normalized using Kappa library quantification kit prior to sequencing. A single pooled library was sequenced as 40bp paired-end dual indexed reads using the high-output (75 cycle) kit on the NextSeq 500 from Illumina. Two C1 runs were performed for 0 and 24-hour single-cell ATAC-seq experiments.

### Single-cell RNA-seq data processing

Single-cell RNA-seq libraries were mapped with Salmon [55] to the mouse Ensembl gene annotations and mm10 reference genome. Single-cell libraries with a mapping rate less than 50% and less than 450,000 mapped reads were excluded from any downstream analysis. Analysis was performed using 0 and 24-hour single-cells.

### Bulk and single-cell ATAC-seq data processing

Single-cell libraries were mapped with Bowtie2 [56] to the mm10 reference genome using the following parameters (bowtie2 -S -p 10—trim3 10 -X 2000). Duplicate fragments were removed using Picard (http://picard.sourceforge.net) as previously performed [8]. We considered single-cell libraries that recovered > 5k fragments after mapping and duplication removal. Bulk ATAC-seq replicates were mapped to the mm10 reference genome using the following parameters (bowtie2 -S—trim3 10 -p 32 -X 2000). Peak calling was performed on bulk replicates using HOMER with the following parameters (findPeaks <tags> -o <output> -localSize 50000 -size 150 -minDist 50 –fragLength 0). The intersection of peaks in three biological replicates was performed. A consolidated list of peaks was generated from the union of peaks from 0 and 24 hour time-points.

### ChIP-seq analysis

*Ikzf1* ChIP-seq data for 0 and 24-hour pre-B cells [29] was mapped to the mm10 reference genome using Bowtie2 [56]. For all samples, we filtered duplicated reads and those with a mapping quality score below 20. To identify peaks, we used the CLCbio Peak Finder software_ENREF_38 [57] with default parameters and control input libraries. We defined significant peaks with an adjusted p-value <0.01 also using biological replicates.

### Training and metaclustering of the individual RNA and ATAC SOMs

We use the SOMatic package, which is a combination of tools written in C++ and R designed for the analysis and visualization of multidimensional genomic or gene expression data, to train our individual SOMs. The SOMatic package also builds a customized, optional javascript viewer to mine the results visually. Installation information for this package can be found at https://github.com/csjansen/SOMatic.

For the RNA-seq SOM, we built a matrix of 12,380 expressed genes in 128 single cells and we used half the genes (6190) to train a self-organizing map with a toroid topology with size 40x60 with 6,190,000 million time steps (1000 epochs) as previously described [25] to select the best of 100 trials based on lowest fitting error. The entire matrix was used for scoring this best trial to generate the final SOM. The SOMatic website for this SOM can be viewed at http://crick.bio.uci.edu/STATegra/RNASOM/

Similarly, the ATAC-seq data was organized into a matrix consisting of scATAC signal in 227 cells at 25,466 ATAC-seq peaks (from pooled data) and half of the peaks were used to train a SOM with a toroid topology with size 40x60 using 12,733,000 time steps (1000 epochs) as previously described [25]. The best of 100 trials based on lowest fitting error was selected and the entire matrix was used for scoring the final SOM. The SOMatic website for this SOM can be viewed at http://crick.bio.uci.edu/STATegra/ATACSOM/

SOM units with similar profiles across cells were grouped into metaclusters [24,25] using SOMatic. Briefly, continuity-constrained [23] metaclustering was performed using k-means clustering to determine centroids for groups of units. Metaclusters were built around these centroids so that each cluster is in one piece to maintain the SOM topology. SOMatic’s metaclustering function attempts all metacluster numbers within a range given and scores them based on Akaike information criterion (AIC) [58]. The penalty term for this score is calculated using a parameter called the “dimensionality,” which is the number of independent dimensions in the data. We performed a hierarchical clustering on the SOM unti vectors and counted the number of clusters that were present at a height level equal to 30% of the total distance in the clustering. For the ATAC-seq SOM, the dimensionality was calculated to be 35, and for the RNA-seq SOM, the dimensionality was calculated to be 128.

For the RNA metaclustering, we tried all metaclusters numbers (k) between 20 and 50, whereas for the ATAC metaclustering we tried all k between 80 and 120. The k with the lowest AIC score was the one chosen for each SOM. For ATAC-seq, 107 metaclusters had the best score, and for RNA-seq, 39 metaclusters had the best score. R scripts for generating metacluster reports are provided in the SOMatic package. Metatcluster/Trait correlation and hypothesis testing analysis were done as previously described [24].

### Hyperparameter variation

There are inherent trade-offs that have to be kept in mind when choosing SOM parameters for training and metaclustering. For example, the size of a SOM is typically one of the most important decisions to be made in analyses of this type. A smaller SOM may group elements together that do not belong together and will reduce the statistical power of down-stream analysis, and a larger SOM may separate elements that belong in the same cluster but are separated due to noise, causing down-stream analysis to miss patterns that may exist. Similarly, the number of timesteps and the learning rate will change the chances of under and over-clustering by changing how the SOM scaffold morphs into the topology of the data. Proper metaclustering can improve the robustness of the SOM by easily revealing improper training due to poor parameters.

The scRNA-seq SOM was built with additional sizes 20x30 and 80x120 with little change to the calculated number of metaclusters, with 36 and 43 respectively. The 20x30 SOM was not chosen for the final analysis due to the occurrence of multiple 1-unit metaclusters, which indicates an underclustering. The 80x120 SOM was not chosen due to having a metacluster that contained a unit in each row which indicated a possible overclustering. The number of timesteps and learning rate chosen were determined to be sufficient due to the smoothness of the final summary map (S6A Fig). An insufficient value in either of these parameters would cause the summary to have large breaks in total signal between neighboring units, indicating under-training.

The scATAC-seq SOM was also built with sizes 20x30, and 80x120 with little change to the calculated number of metaclusters, with 98 and 109 respectively. The 80x120 SOM was not chosen due to the map focusing too much on regions that were unique to each cell, indicating overclustering. The 40x60 size was chosen over the 20x30 due to it having a better score. The number of timesteps and learning rate chosen were determined to be sufficient due to the smoothness of the final summary map (S6B Fig).

### Linked SOMs

In order to define this, a few preliminary definitions are required. For a set *A* of data vectors, it is possible to define a set of *n* vectors, *B*, indexed on a 2D lattice to partition *A* into *n* subsets with each vector assigned to the subset *i* iff *B*_*i*_ is the closest element of *B* to that vector. Due to the 2D indexing lattice that they are placed on, each vector in *B* is adjacent to its closest member in *B*, with “closest” defined by an unsupervised neural network. The set of vectors, *B*, is the set of SOM units.

Similarly, it is possible to compute a set of *m* vectors, *M*, to partition *B* into *m* subsets, *S*, with each vector assigned to the subset *i* iff *M*_*i*_ is the closest element in *M* to that vector such that a path can be drawn on the lattice using only elements of *S*_*i*_. This path requirement is in place to maintain the SOM topology calculated in training of the neural network. The subsets, *S*, are the metaclusters defined previously.

Let *G* be the set of gene vectors from a number of RNA-seq experiments and let *R* be the set of genome region vectors defined by ATAC-seq peaks. Using the procedure above, it is possible to segment these sets into metaclusters, named *N* and *M* respectively. Between these two metacluster partitions, we can define a linker mapping, *h*, from *R* to *G*. Using a linker mapping designed to link the individual SOM datatypes, we can define a set of partitions, *F*^*M*,*N*,*h*^, where (*r*,*g*) ϵ (*R*,*G*) is an element of *F*^*M*,*N*,*h*^_*ij*_ iff *h*(*r*) = *g*, *g* ϵ *N*_*j*_, and *r* ϵ *M*_*i*_. In this case, the linker mapping that we use to link RNA and open chromatin data is an implementation of the GREAT [34] OneClosest algorithm with a cutoff of 50kb to build regulatory regions around transcription start sites for each gene and check if these regions overlap with the ATAC-seq peaks. The resulting Linked SOM metaclusters (LMs) contain clusters of similar genome regions such that their linked genes are also similar.

### Motif analysis

The regulatory regions, including repeat regions, in each Linked SOM metacluster were separately scanned for motifs from the HOCOMOCOv11 mouse motif database [59] with FIMO v4.12.0 [60] using a q-value threshold of .05. The background for FIMO was calculated using the entire mm10 reference genome. Then, for each transcription factor in the database, the percentage of regions in each LM with a motif for that factor was calculated. To determine enrichment, the percentages for each transcript factor were separately compared in a one-tailed z-score analysis. LMs with a percentage that was significantly (pvalue < .05) enriched over the baseline, the average percentage across all LMs for that transcription factor motif, was reported for each transcription factor. Finally, transcription factors with a statistically significant number of motifs were mapped to the gene fused to the regulatory region the motif was found within. The full list of these potential connections can be found here: http://crick.bio.uci.edu/STATegra/LinkedMotifMappings.txt.

### sciCar Scalability analysis and cisTopic scATAC-seq Analysis

Processed sciCar data was downloaded from NCBI GEO (GSE117089) and reformatted into data matrices for both the scRNA-seq and scATAC-seq data. Of the ~4825 cells with measurements in both experiments, we kept those (3,234 cells) with more than 5% of genes detected in the RNA signal and more than 1000 mapped fragments detected in the ATAC signal. Additionally, we removed genes that were detected in less than 5% of cells and peaks with less than 100 total reads in all cells that passed the above filter. In total, the final RNA matrix was 17,751 genes x 3,234 cells and the final ATAC matrix was 18,638 peaks x 3,234 cells. These matrices were both trained into 40x60 SOMs over 1000 epochs with 100 replicates (best score taken) as above. We then performed the entire Linked SOMs pipeline above using the hg19 reference genome and Homo_sapiens.GRCh37.87 gene annotations. Finally, we ran the SOMatic pipeline on subsets of this data and recorded the runtime (S23 Fig). This run was on an AMD EPYC 7601 32-Core Processor with 2,200 MHz and 5GiB of RAM.

For the cisTopic analysis, we input the pre-B cell ATAC-seq training matrix into cisTopic v0.2.0 and followed the vignette on their GitHub. (https://github.com/aertslab/cisTopic)

### GEO accession

GEO accession number for data is GSE89285.

## Supporting information

S1 Fig Self-Organizing map clustering overview. (A) Example heatmap for 5 genes’ expression in a typical single-cell RNA-seq with 2 time points. Genes G1 and G2 are enriched at 0h with two 0h cells missing that signal due to technical noise and gene G4 is enriched at 24hr. Genes G3 and G5 also have a similar expression pattern with two cells missing signal in G5 due to technical noise, but are not particularly enriched in either time point. (B) 2D representation of the genes’ expression profile with an initial SOM scaffold. The colors in the scaffold correspond to those the map below. (C) 2D representation of the genes’ expression profile with a typical trained SOM scaffold overlaid. The maps below represent the signal for each unit in the labeled experiment’s dimension. For example, only gene G4 has signal in 24h Cell #1, and thus, only the unit near G4 has signal on the map. (D) Neighboring units with similar expression profiles are metaclustered to fix the overclustering of genes G1 and G2 into separate units. (E) Multiple individual maps can be combined into one through arithmetic. This map represents the average of each 24h map subtracted from the average of each 0h map. (F) Trait enrichment analysis can be applied on each metacluster to provide a p-value for enrichment in a particular time point. Here, metacluster 1, containing genes G1 and G2, is enriched in 0h, and metacluster 3, containing gene G3, is enriched in 24h.
(TIF)

S2 Fig scRNA-seq gene UMAP. UMAP [30] generated using uwat [61] from scRNA-seq data with each point representing a gene’s expression in each cell. The umap is separated into 4 large clusters, which provides a poor level of resolution for downstream analysis. Points were colored by RNA SOM metacluster, which divides the large clusters into many sub-clusters.
(TIF)

S3 Fig scATAC-seq region UMAP. UMAP [30] generated using uwat [61] from scATAC-seq data with each point representing a genome region’s ATAC-seq signal in each cell. The umap could not be separated into any significant clusters. Points were colored by ATAC SOM metacluster, which divides the large cluster into many sub-clusters.
(TIF)

S4 Fig UMAPs of cells used in analysis. UMAP [30] generated using Seurat v3 [31] from both data types with each point representing a cell colored by timepoint.
(TIF)

S5 Fig Seurat v3 co-embedding. Co-embedding of the scRNA-seq and scATAC-seq data created using Seurat v3 [31]. The co-embedding succeeded in overlapping cells from the different technologies, but failed to use the clear separation of the time points in the scRNA-seq data to separate the co-embedding by time.
(TIF)

S6 Fig SOM summary maps (total signal in every cell). (A-B) Summary maps for the (A) RNA and (B) ATAC SOMs. Each unit’s value is generated by totaling the values in the full SOM unit’s vector. A blue-white-red color spectrum was used. These graphs are mainly used to determine ‘smoothness’ of the SOM fit and to see if more timesteps or changes to the learning rate are needed.
(TIF)

S7 Fig Statistic maps for scRNA-seq SOM. (A) U-Matrix for the SOM built with the single-cell RNA-seq dataset. Each unit contains the average of the distance to all neighboring units. Metacluster divisions are overlaid. Areas of high distance correspond primarily to a metacluster division. (B) Density map for the RNA-seq SOM. The color corresponds to the number of genes found in each unit. Metacluster divisions are overlaid. Most metaclusters are ruled by a few high density units.
(TIF)

S8 Fig Statistic maps for scATAC-seq SOM. (A) U-Matrix for the SOM built with the single-cell ATAC-seq dataset. Each unit contains the average of the distance to all neighboring units. Metacluster divisions are overlaid. Areas of high distance correspond primarily to a metacluster division. (B) Density map for the ATAC-seq SOM. The color corresponds to the number of chromatin regions found in each unit. Metacluster divisions are overlaid. Most metaclusters are ruled by a few high density units.
(TIF)

S9 Fig cisTopic Analysis of Pre-B cell ATAC-seq Data. (A) Graph detailing the score of various topics tried in cisTopic training. The best model had 15 topics. (B) T-sne output from cisTopic after training. Each point is a cell colored by timepoint (Yellow is 0 hr and green is 24 hr). (C) Bar graph detailing the number of regions in each called topic. (D) Comparison of cisTopic topics and SOM linked metaclusters. Several ATAC-seq peaks with very different profiles ended up in different ATAC-seq SOM metaclusters and the same cisTopic topic.
(TIF)

S10 Fig Global Topic/Metacluster comparison heatmap. (A) Heatmap of Topic/Metacluster overlap normalized by Metacluster ordered by hierarchical clustering. (B) Heatmap of the absolute number of regions in each Topic/Metacluster overlap ordered by hierarchical clustering.
(TIF)

S11 Fig Silhouette coefficient comparison between cisTopic and SOM Metaclusters. (A-B) Silhouette coefficient graphs for the cisTopic and SOMatic clusterings of the mouse pre-B cell scATAC-seq data. The average Silhouettes were very similar despite cisTopic only using 8,266 of the 25,466 genomic regions.
(TIF)

S12 Fig SOM Linking overview. (A) An example SOM after training on RNA-seq data. Metaclusters 1, 2, and 3 contain genes (G1, G2), (G3, G5), and (G4) respectively. (B) An example SOM after training on ATAC-seq data. Metaclusters 1, 2, and 3 contain genome regions (R3, R5), (R1, R2, R7), and (R4, R6) respectively. (C) An example of how the genes in (a) and the genome regions in (b) could be arranged with their respective metaclusters. (D) The final list of linked metaclusters (LM) that result from the above system. Note that Region 1 and 2 both end up in the same LM (ATAC 2, RNA 1) because they are both in ATAC metacluster 2 and their nearby genes, G1 and G2, are both in RNA metaclusters 1. (E) Example motif enrichments for each gene in (A) in each LM. Bolded genes have a significant enrichment over the background. G1 is found too highly in many LMs and might have an extremely permissive motif. In LM (ATAC 1, RNA 3), G3 motif is found, but would not be called significant due to it being only 1 observation. (F) An example gene regulatory network generated from (E).
(TIF)

S13 Fig Motif mining efficiency using various techniques. (A) Graph detailing the number of motifs found using the same set of peaks with different groupings using the same q-value < .05 cutoff. (B) Graph detailing the number of motifs found using the same set of peaks with using the linked metacluster grouping and just the ATAC-seq SOM metaclusters grouping using the same q-value < .05 cutoff.
(TIF)

S14 Fig Motif scanning statistics for random separation validation. The distribution of motifs found by randomly splitting peaks into 4,429 synthetic linked metaclusters (LM). The mean was ~1,469,000 motifs which is significantly fewer than the ~9.3 million found in the real LMs.
(TIF)

S15 Fig Chromatin accessibility patterns around Igll1 and Vpreb1 locus revealed by scATAC-seq labeled by SOMatic. (A-B) UCSC genome browser screenshots of the *Igll1* and *Vpreb1* loci with bulk (50,000 cells), aggregate (94 single-cells averaged) and single-cell ATAC-seq for 0 (A; 94 single-cells) and 24-hour (B;133 single-cells) pre-B cells. Linked SOM ids (ATAC, RNA) are depicted for all chromatin elements.
(TIF)

S16 Fig ChIP-seq validation of Ikaraos binding near Nr3c1. UCSC genome browser snapshots of Ikaros ChIP data taken at the 0-hour and 24-hour timepoints near *Nr3c1*. The location of the predicted motif is noted along with its linked metacluster ID. The marked location has a significant change in binding at the marked location over the time course.
(TIF)

S17 Fig ChIP-seq validation of Ikaraos binding near *Elf2*. UCSC genome browser snapshots of Ikaros ChIP data taken at the 0-hour and 24-hour timepoints near *Elf2*. There were 2 predicted motifs in this metacluster, Ikaros and Tcf3.
(TIF)

S18 Fig ChIP-seq validation of Ikaraos binding near *Hes1*. UCSC genome browser snapshots of Ikaros ChIP data taken at the 0-hour and 24-hour timepoints near *Hes1*. The location of the predicted motif is noted along with its linked metacluster ID. The marked location has a significant change in binding at the marked location over the time course.
(TIF)

S19 Fig Downstream *Myc* target gene expression and chromatin accessibility dynamics. Myc (whose signal drops dramatically from 0- to 24- hour) downstream targets were predicted in a method similar to that in Fig 4. Around half of these react with a drop in signal with a small portion reacting with an increase. This is similar to the change in chromatin accessibility at the predicted binding sites near these genes.
(TIF)

S20 Fig Gene regulatory connections downstream of Ikaros with levels of known evidence. A list of transcription factors with significant changes over the time course and the transcription factors were predicted to regulate. Each regulated gene is followed by a label for the level of existing evidence and reference number if relevant.
(TIF)

S21 Fig Application of linked metaclusters on sciCar data. (A-B) Difference maps displaying the areas of temporal enrichment after training on sciCar data. (C) RNA data was differential in 19 metaclusters that represent 2113 genes. The ATAC data was differential in 11 metaclusters representing 2607 genome regions. (D) Violin plots describing the distribution of average temporal correlations between linked ATAC-seq peaks and genes. The differential metaclusters (in green) have fewer combinations with no correlation and more with negative correlations than the distributions from all LMs (in red). Both distributions are significantly (pvalue < .05) different than when the timepoints of the cells are scrambled (in blue). (E) A heatmap of the average temporal correlations from the differential linked metaclusters. (F) Known targets of Nr3c1 (GR receptor) recovered during motif and network analysis. These downstream genes all appeared in differential RNA metaclusters.
(TIF)

S22 Fig Comparison of linked metacluster profiles to scaled profiles from sciCar data. (A) Re-computed pseudotime graph of the scRNA-seq data from the sciCar dataset by monocle [18] is very similar to original publication. (B-C) The scaled signal of the gene expression for ZSWIM6 and chromatin accessibility for its promoter from binned cells is very similar to the original publication. (D) Violin plots of the computed correlations between gene expression and their promoters’ accessibility. Linked metacluster profiles had significantly higher correlations than simply scaling the profiles.
(TIF)

S23 Fig SOMatic runtime graph on sciCar scATAC-seq data. Runtime estimates for SOMatic running on different number of samples. Online SOMs have an inherent complexity of O(Observations*Samples) which SOMatic replicates.
(TIF)
