# Supplementary figures and images for: Building gene regulatory networks from scATAC-seq and scRNA-seq using Linked Self Organizing Maps
Source: PLoS Comput Biol. 2019 Nov 4;15(11):e1006555. doi: 10.1371/journal.pcbi.1006555 (PMC6855564; doi:10.1371/journal.pcbi.1006555)

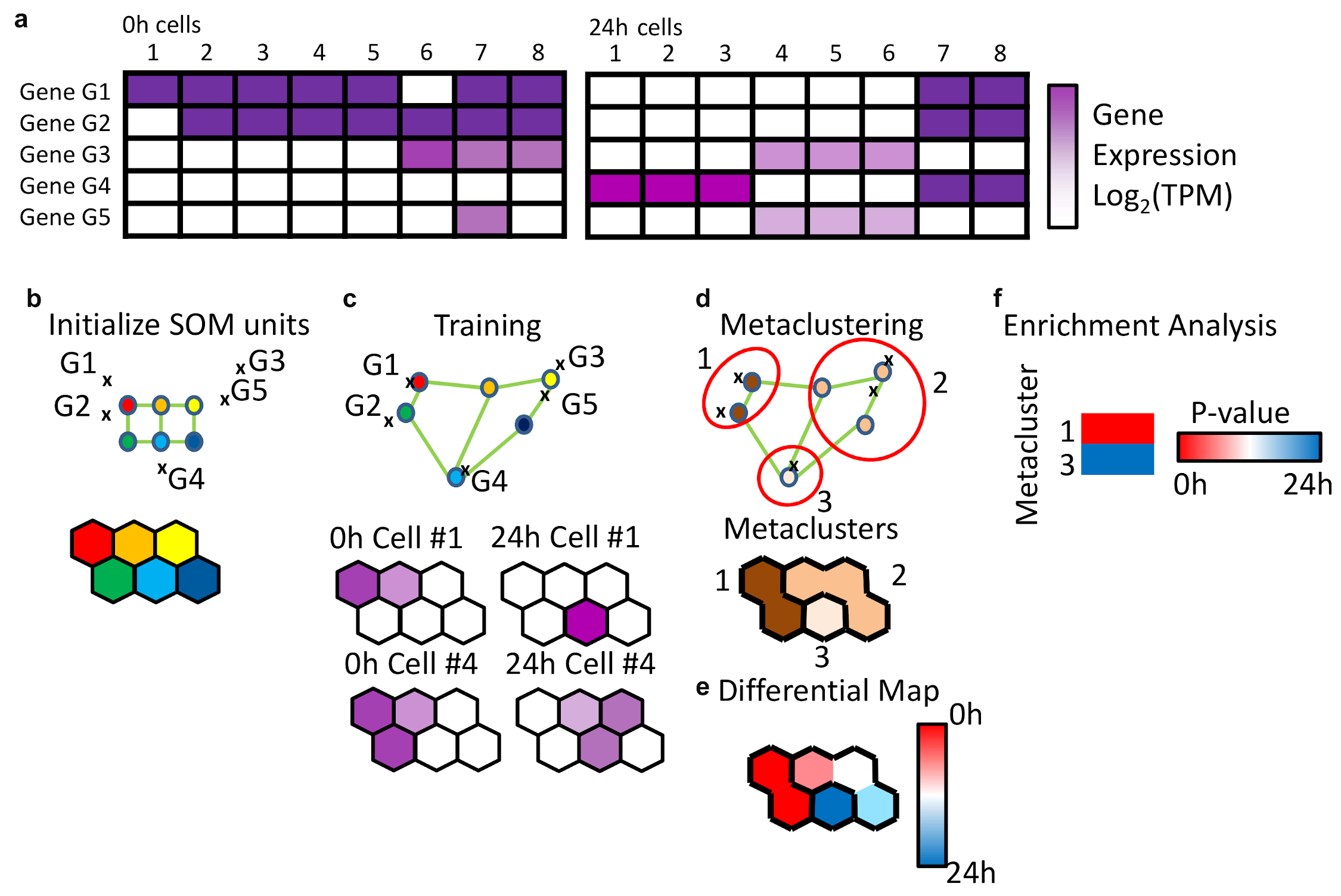

Supplement: S1 Fig — (A) Example heatmap for 5 genes’ expression in a typical single-cell RNA-seq with 2 time points. Genes G1 and G2 are enriched at 0h with two 0h cells missing that signal due to technical noise and gene G4 is enriched at 24hr. Genes G3 and G5 also have a similar expression pattern with two cells missing signal in G5 due to technical noise, but are not particularly enriched in either time point. (B) 2D representation of the genes’ expression profile with an initial SOM scaffold. The colors in the scaffold correspond to those the map below. (C) 2D representation of the genes’ expression profile with a typical trained SOM scaffold overlaid. The maps below represent the signal for each unit in the labeled experiment’s dimension. For example, only gene G4 has signal in 24h Cell #1, and thus, only the unit near G4 has signal on the map. (D) Neighboring units with similar expression profiles are metaclustered to fix the overclustering of genes G1 and G2 into separate units. (E) Multiple individual maps can be combined into one through arithmetic. This map represents the average of each 24h map subtracted from the average of each 0h map. (F) Trait enrichment analysis can be applied on each metacluster to provide a p-value for enrichment in a particular time point. Here, metacluster 1, containing genes G1 and G2, is enriched in 0h, and metacluster 3, containing gene G3, is enriched in 24h. (TIF) [file pcbi.1006555.s001.tif]

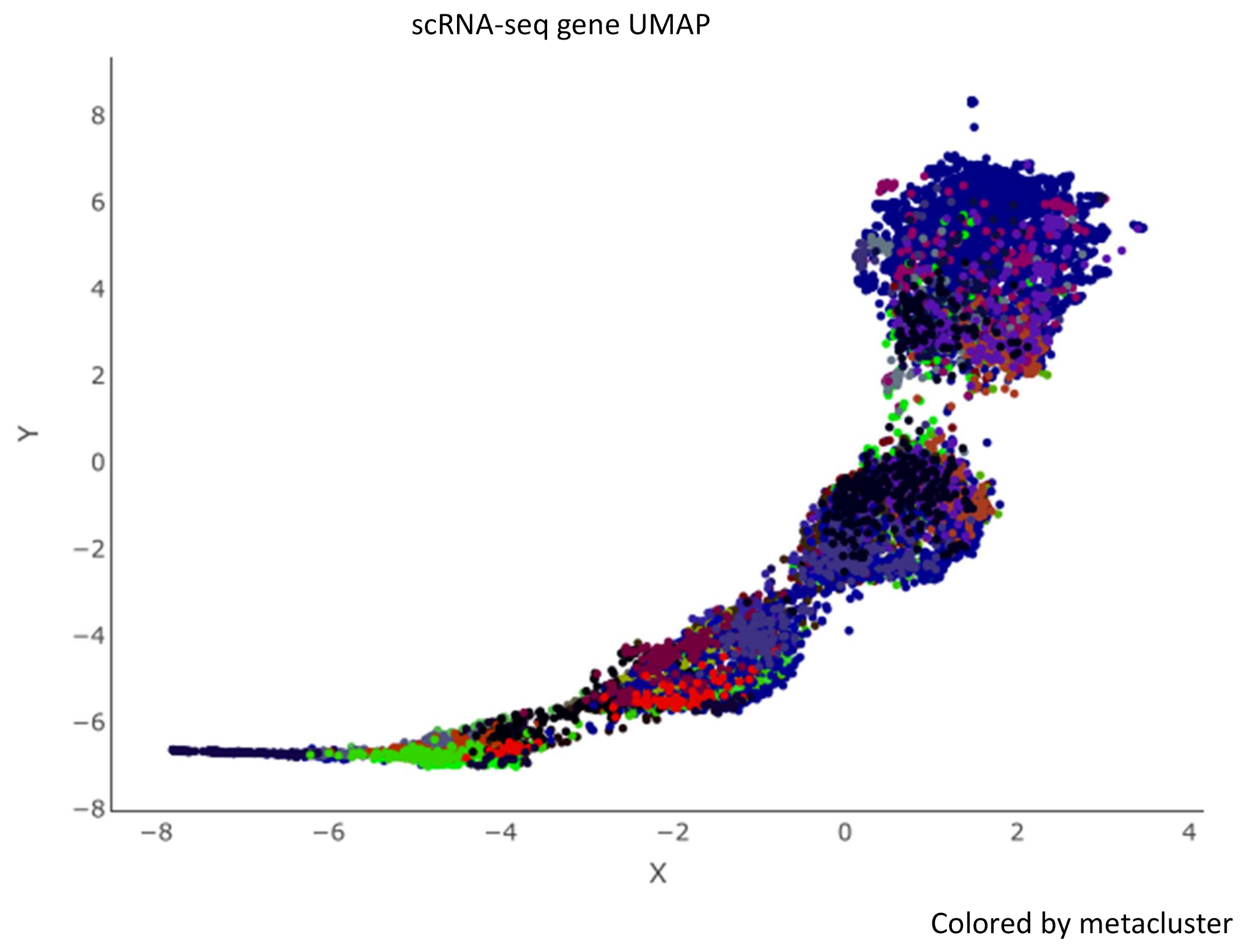

Supplement: S2 Fig — UMAP [30] generated using uwat [61] from scRNA-seq data with each point representing a gene’s expression in each cell. The umap is separated into 4 large clusters, which provides a poor level of resolution for downstream analysis. Points were colored by RNA SOM metacluster, which divides the large clusters into many sub-clusters. (TIF) [file pcbi.1006555.s002.tif]

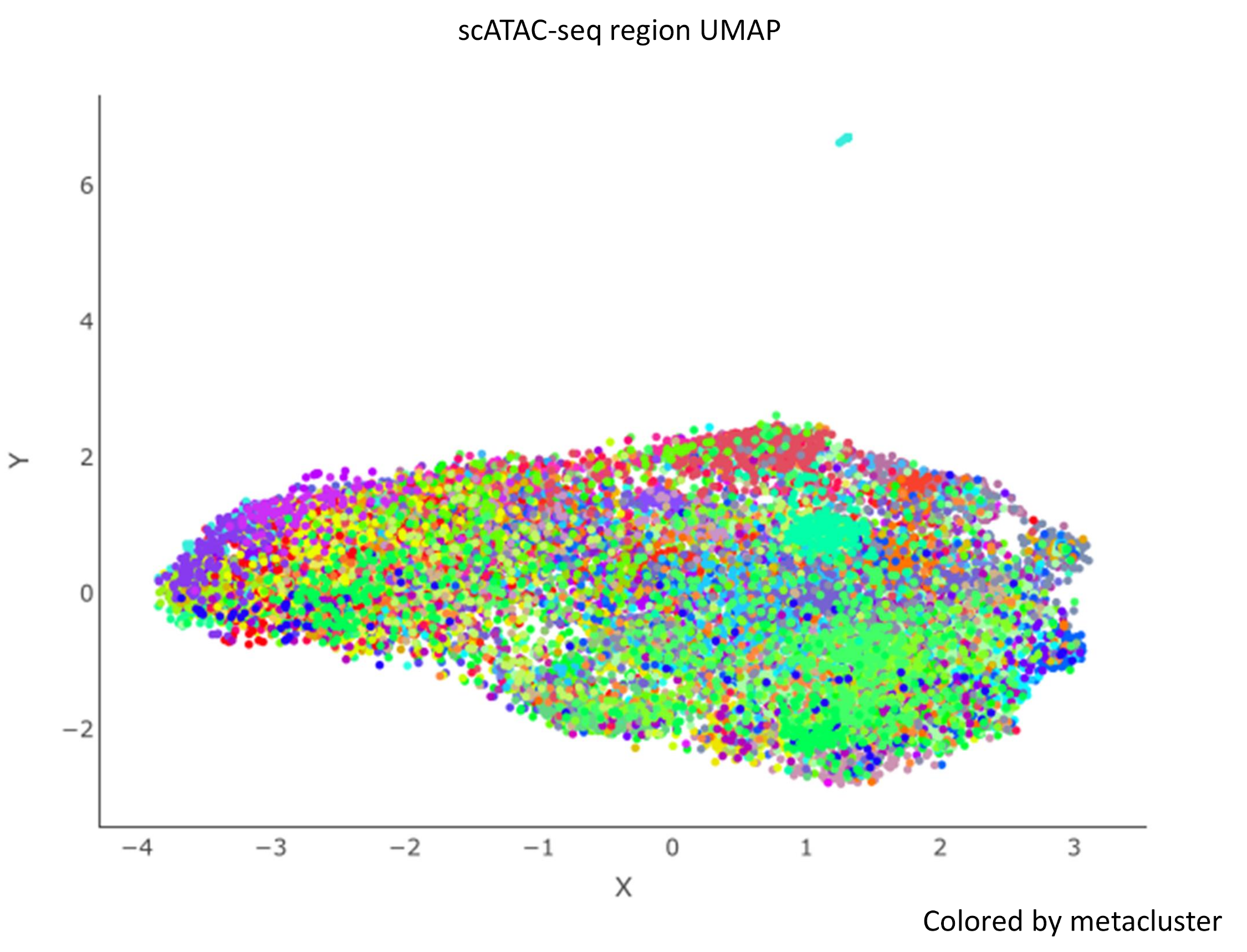

Supplement: S3 Fig — UMAP [30] generated using uwat [61] from scATAC-seq data with each point representing a genome region’s ATAC-seq signal in each cell. The umap could not be separated into any significant clusters. Points were colored by ATAC SOM metacluster, which divides the large cluster into many sub-clusters. (TIF) [file pcbi.1006555.s003.tif]

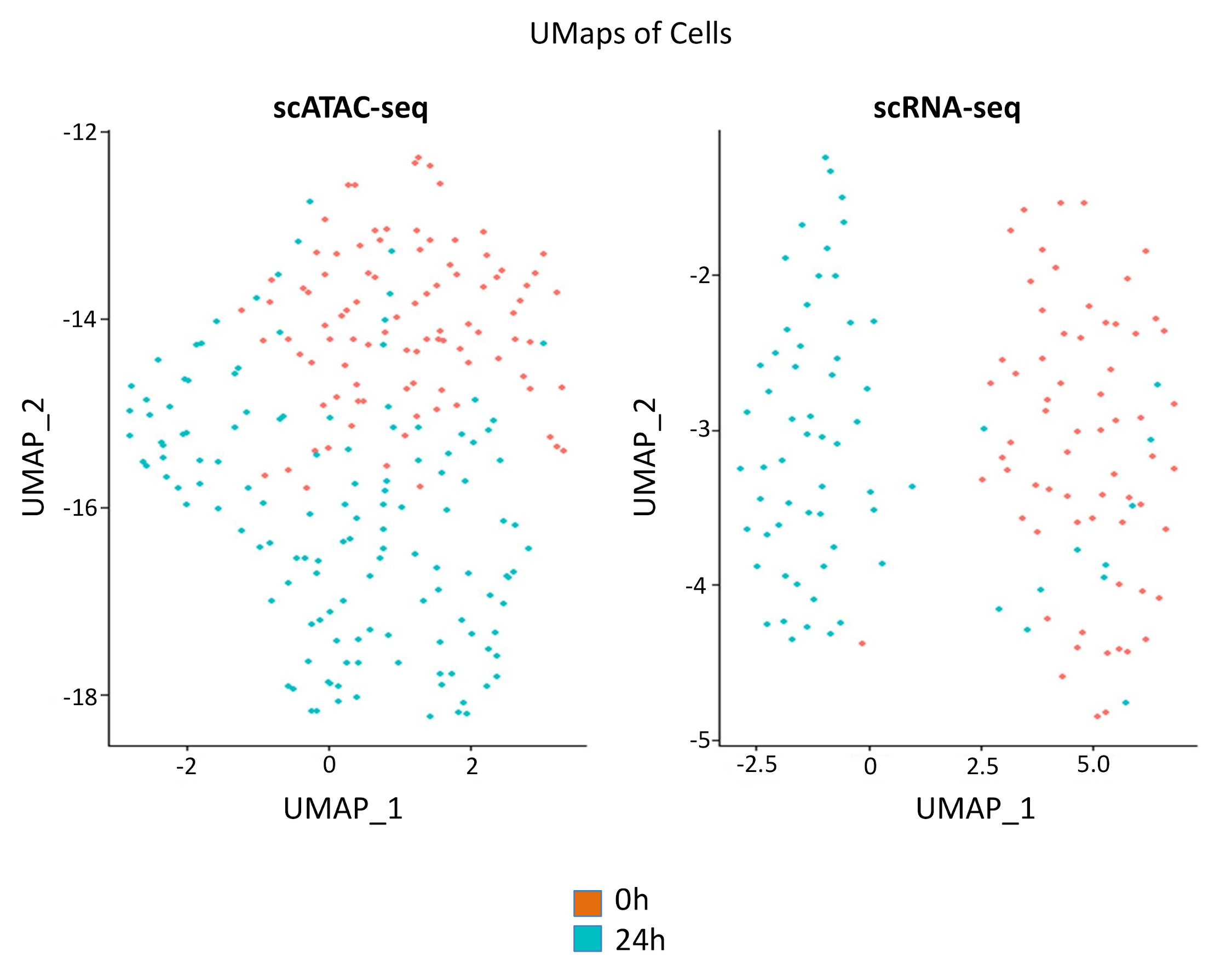

Supplement: S4 Fig — UMAP [30] generated using Seurat v3 [31] from both data types with each point representing a cell colored by timepoint. (TIF) [file pcbi.1006555.s004.tif]

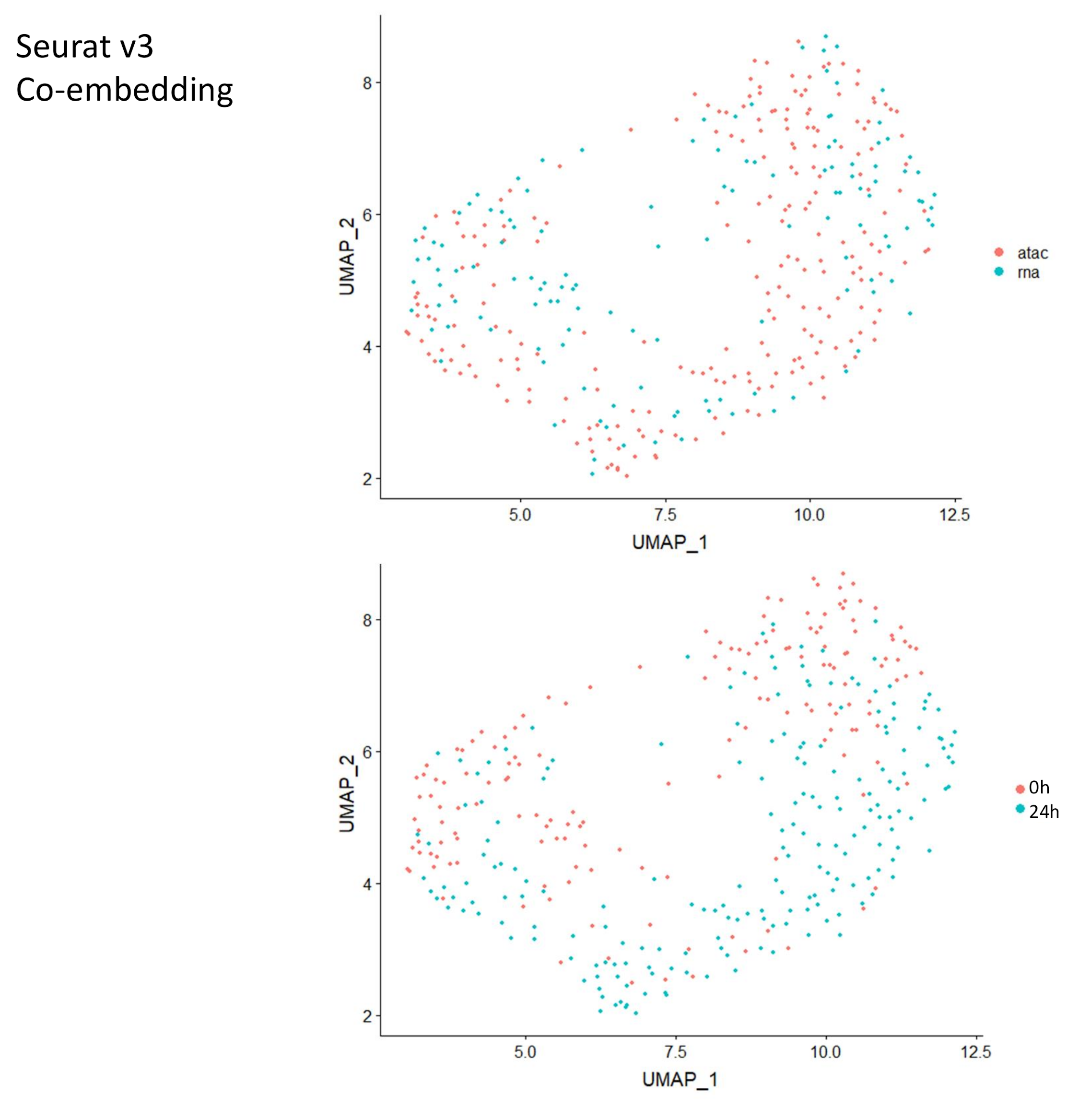

Supplement: S5 Fig — Co-embedding of the scRNA-seq and scATAC-seq data created using Seurat v3 [31]. The co-embedding succeeded in overlapping cells from the different technologies, but failed to use the clear separation of the time points in the scRNA-seq data to separate the co-embedding by time. (TIF) [file pcbi.1006555.s005.tif]

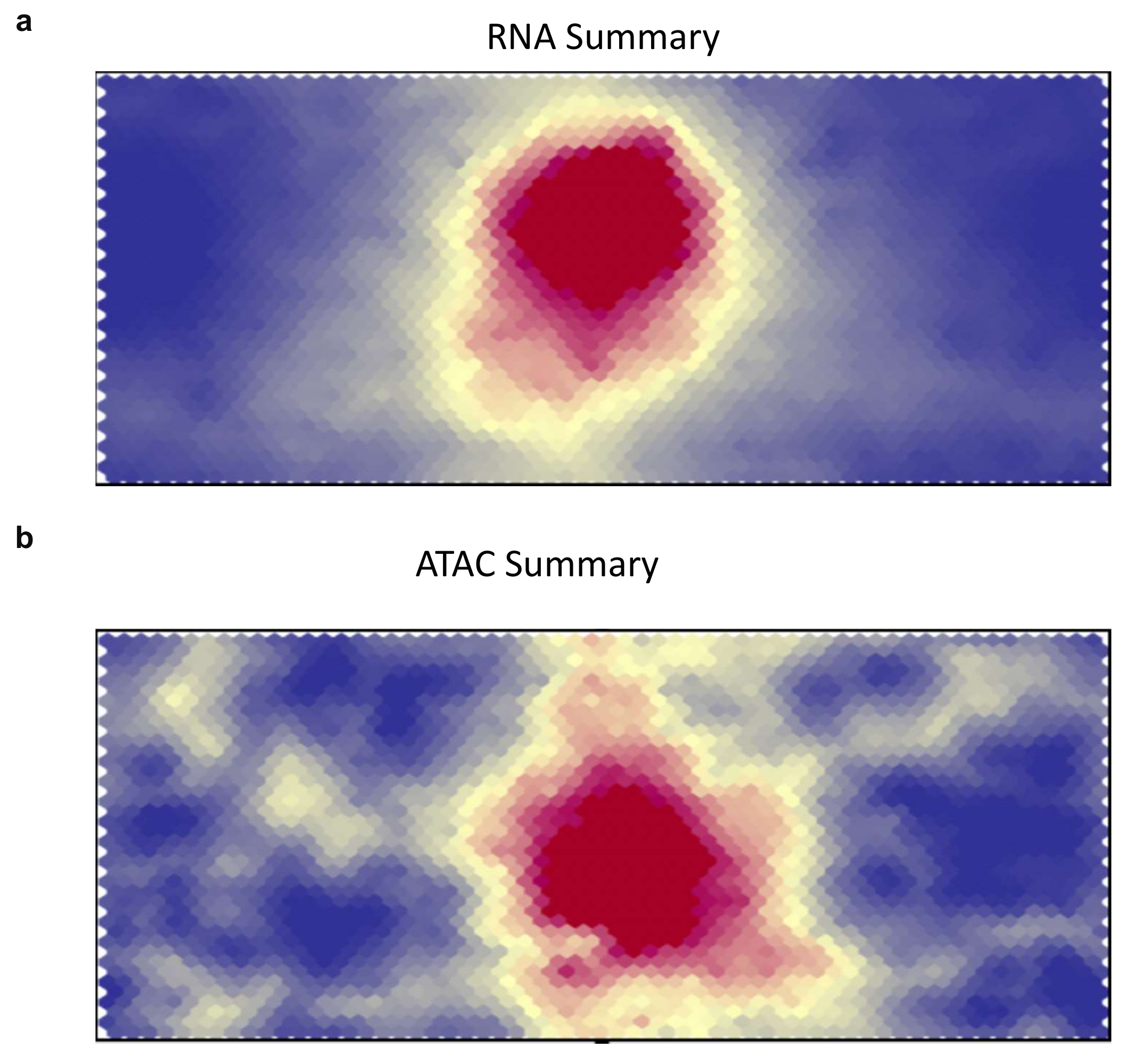

Supplement: S6 Fig — (A-B) Summary maps for the (A) RNA and (B) ATAC SOMs. Each unit’s value is generated by totaling the values in the full SOM unit’s vector. A blue-white-red color spectrum was used. These graphs are mainly used to determine ‘smoothness’ of the SOM fit and to see if more timesteps or changes to the learning rate are needed. (TIF) [file pcbi.1006555.s006.tif]

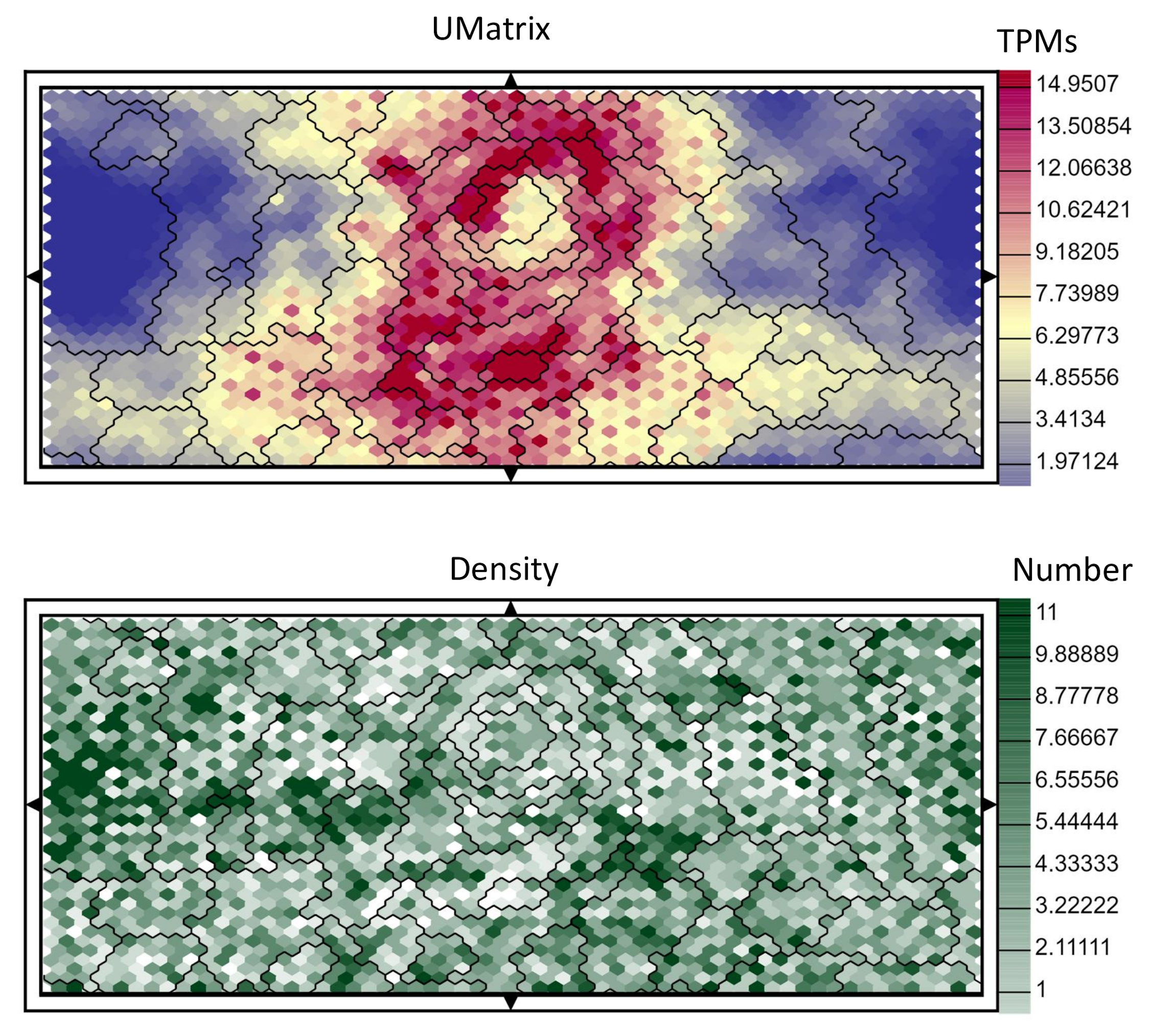

Supplement: S7 Fig — (A) U-Matrix for the SOM built with the single-cell RNA-seq dataset. Each unit contains the average of the distance to all neighboring units. Metacluster divisions are overlaid. Areas of high distance correspond primarily to a metacluster division. (B) Density map for the RNA-seq SOM. The color corresponds to the number of genes found in each unit. Metacluster divisions are overlaid. Most metaclusters are ruled by a few high density units. (TIF) [file pcbi.1006555.s007.tif]

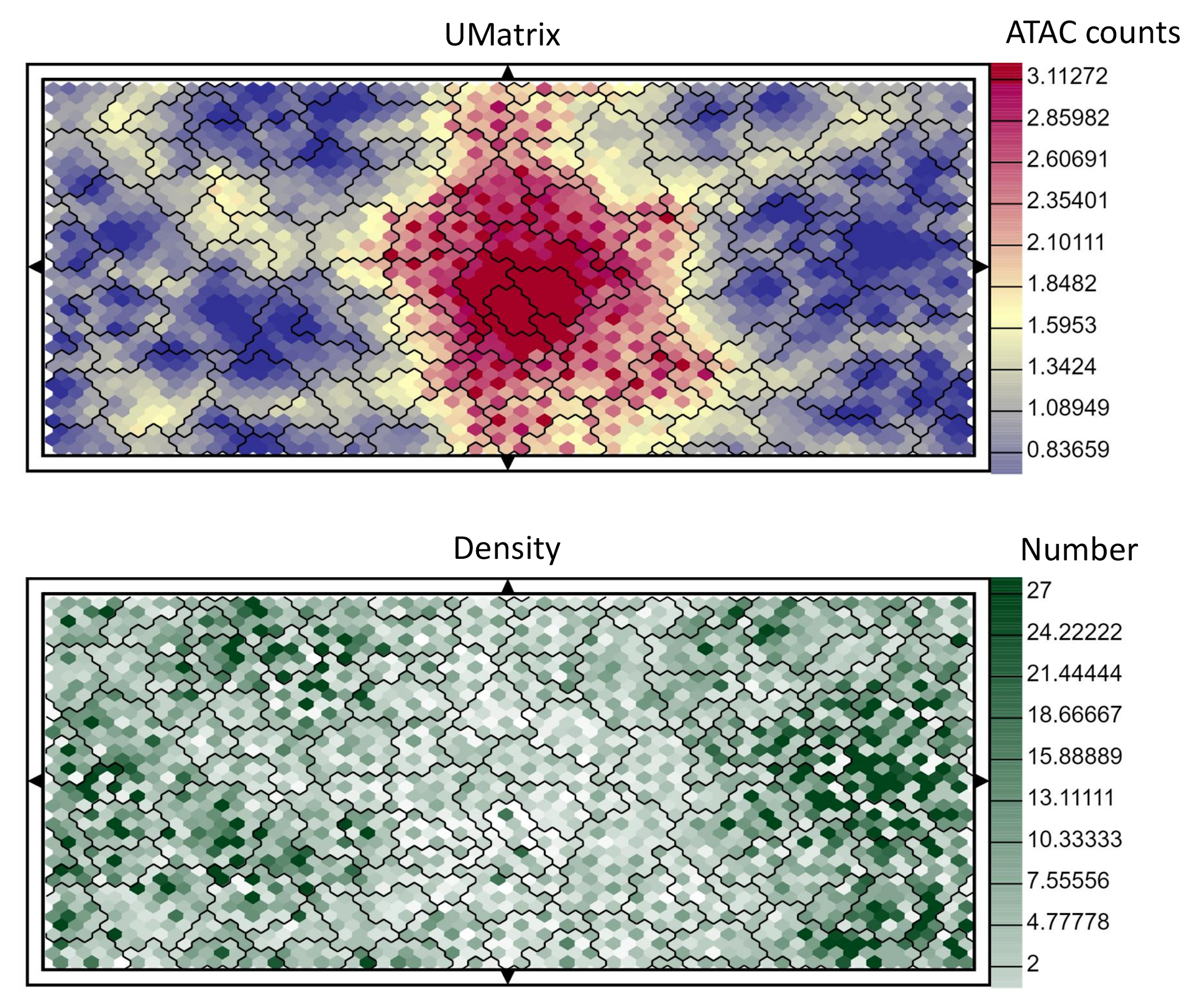

Supplement: S8 Fig — (A) U-Matrix for the SOM built with the single-cell ATAC-seq dataset. Each unit contains the average of the distance to all neighboring units. Metacluster divisions are overlaid. Areas of high distance correspond primarily to a metacluster division. (B) Density map for the ATAC-seq SOM. The color corresponds to the number of chromatin regions found in each unit. Metacluster divisions are overlaid. Most metaclusters are ruled by a few high density units. (TIF) [file pcbi.1006555.s008.tif]

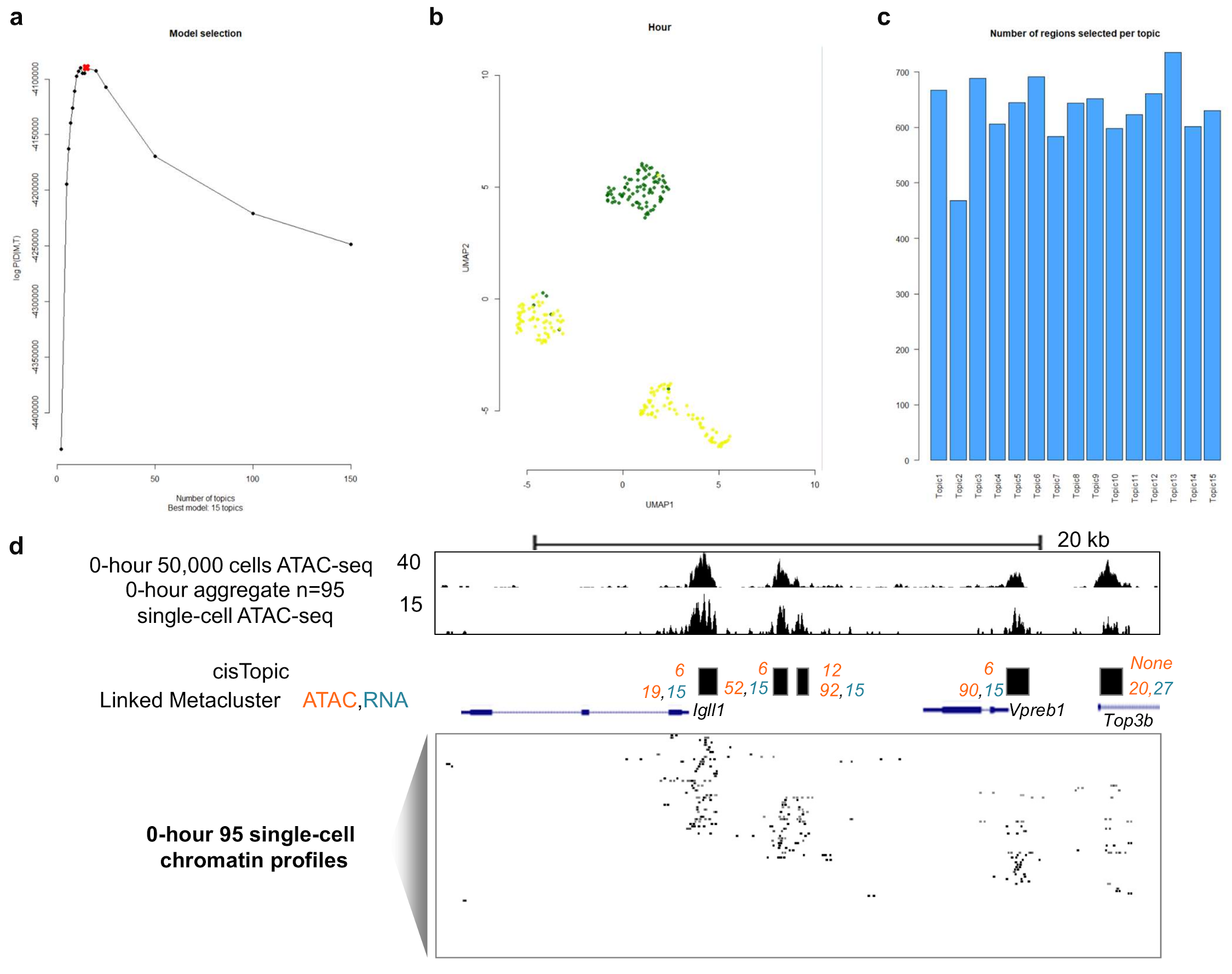

Supplement: S9 Fig — (A) Graph detailing the score of various topics tried in cisTopic training. The best model had 15 topics. (B) T-sne output from cisTopic after training. Each point is a cell colored by timepoint (Yellow is 0 hr and green is 24 hr). (C) Bar graph detailing the number of regions in each called topic. (D) Comparison of cisTopic topics and SOM linked metaclusters. Several ATAC-seq peaks with very different profiles ended up in different ATAC-seq SOM metaclusters and the same cisTopic topic. (TIF) [file pcbi.1006555.s009.tif]

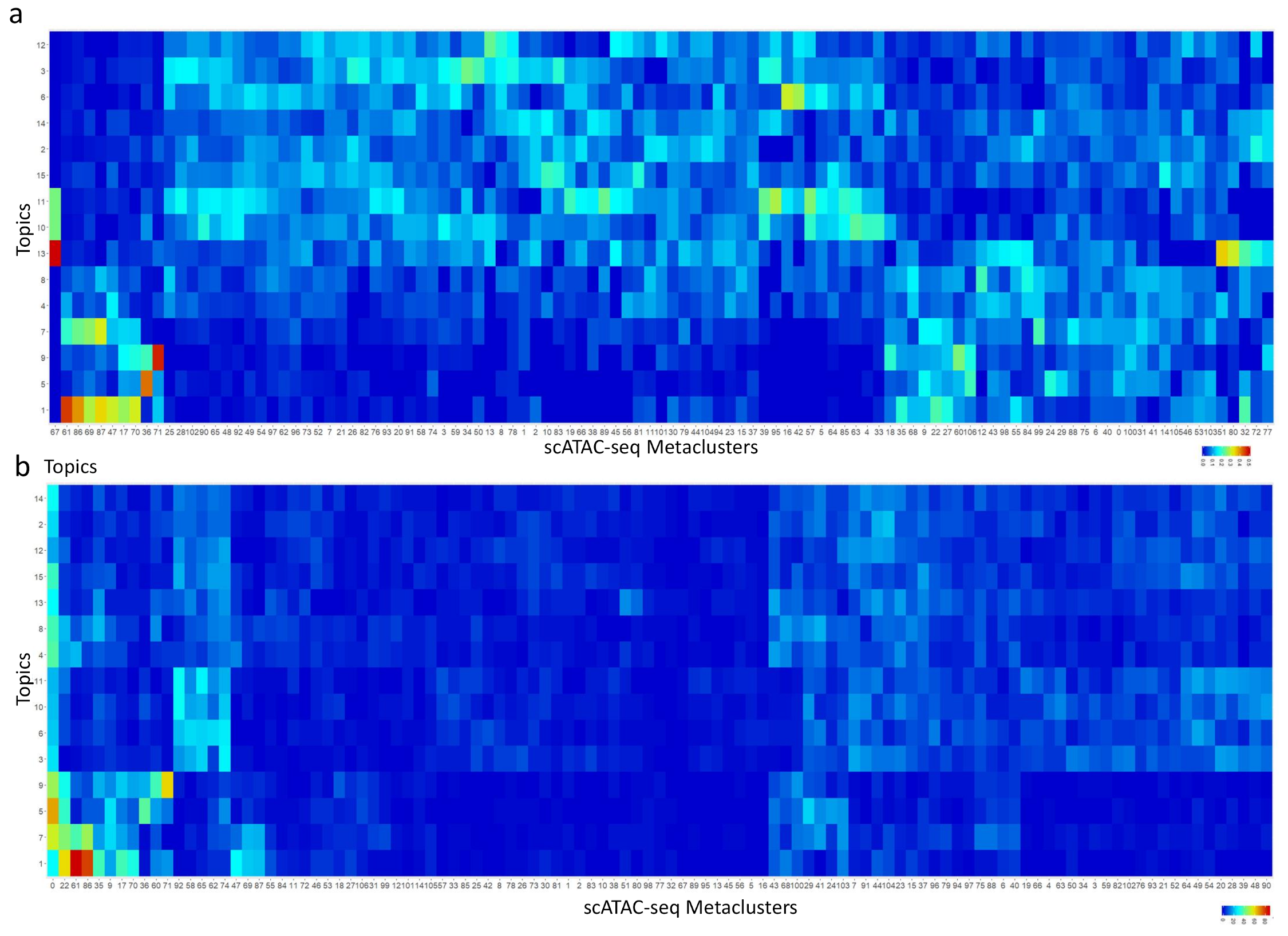

Supplement: S10 Fig — (A) Heatmap of Topic/Metacluster overlap normalized by Metacluster ordered by hierarchical clustering. (B) Heatmap of the absolute number of regions in each Topic/Metacluster overlap ordered by hierarchical clustering. (TIF) [file pcbi.1006555.s010.tif]

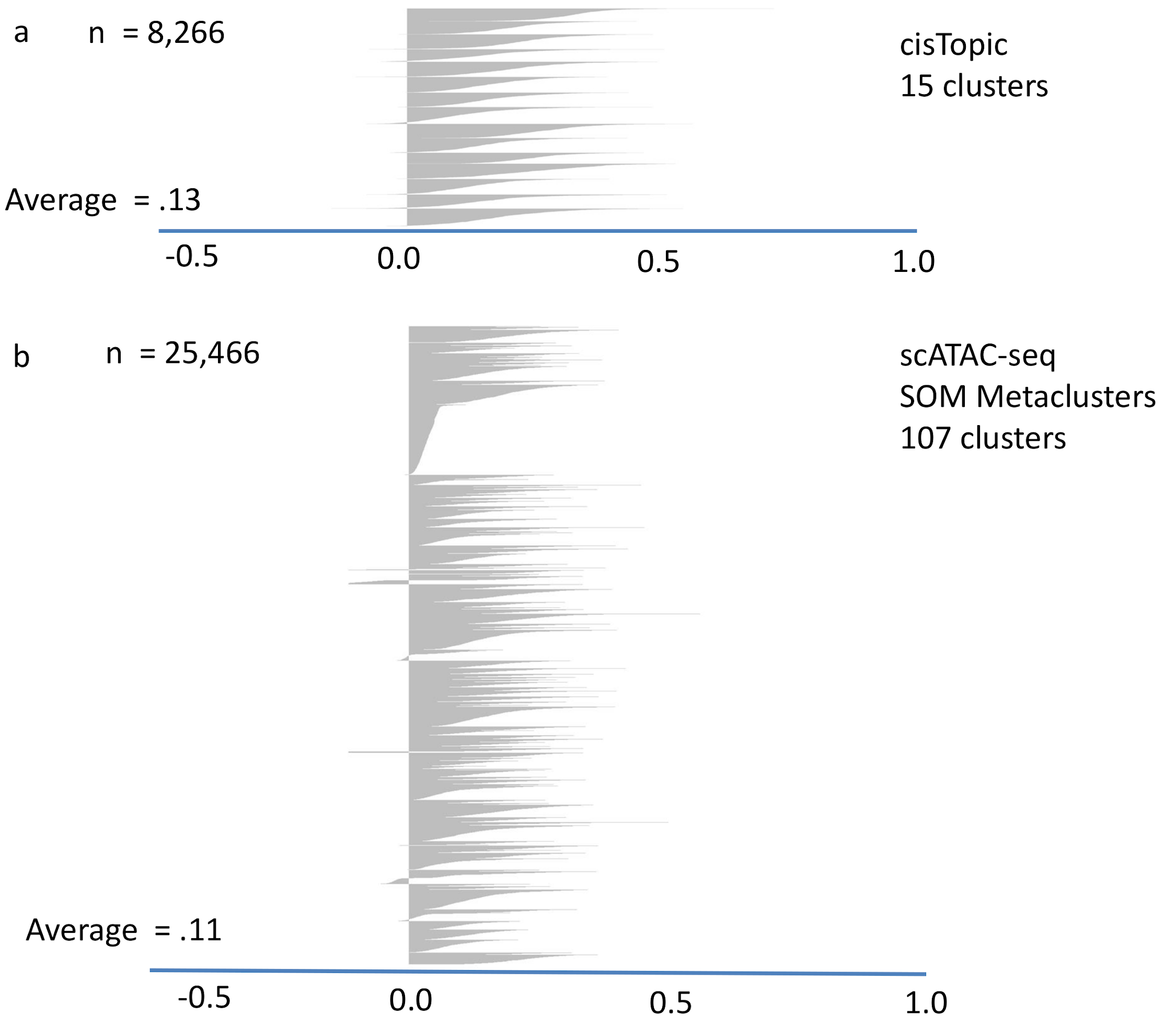

Supplement: S11 Fig — (A-B) Silhouette coefficient graphs for the cisTopic and SOMatic clusterings of the mouse pre-B cell scATAC-seq data. The average Silhouettes were very similar despite cisTopic only using 8,266 of the 25,466 genomic regions. (TIF) [file pcbi.1006555.s011.tif]

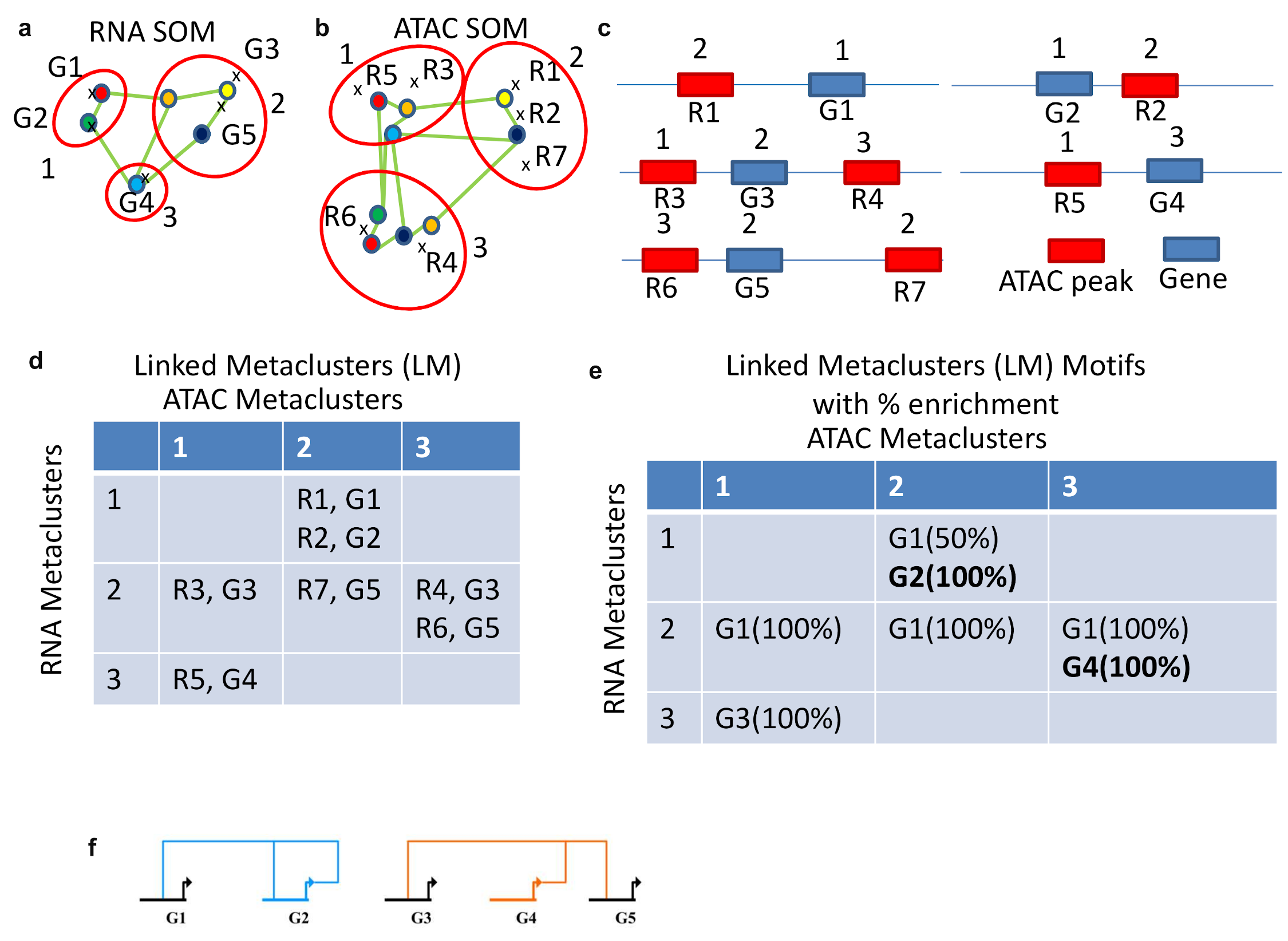

Supplement: S12 Fig — (A) An example SOM after training on RNA-seq data. Metaclusters 1, 2, and 3 contain genes (G1, G2), (G3, G5), and (G4) respectively. (B) An example SOM after training on ATAC-seq data. Metaclusters 1, 2, and 3 contain genome regions (R3, R5), (R1, R2, R7), and (R4, R6) respectively. (C) An example of how the genes in (a) and the genome regions in (b) could be arranged with their respective metaclusters. (D) The final list of linked metaclusters (LM) that result from the above system. Note that Region 1 and 2 both end up in the same LM (ATAC 2, RNA 1) because they are both in ATAC metacluster 2 and their nearby genes, G1 and G2, are both in RNA metaclusters 1. (E) Example motif enrichments for each gene in (A) in each LM. Bolded genes have a significant enrichment over the background. G1 is found too highly in many LMs and might have an extremely permissive motif. In LM (ATAC 1, RNA 3), G3 motif is found, but would not be called significant due to it being only 1 observation. (F) An example gene regulatory network generated from (E). (TIF) [file pcbi.1006555.s012.tif]

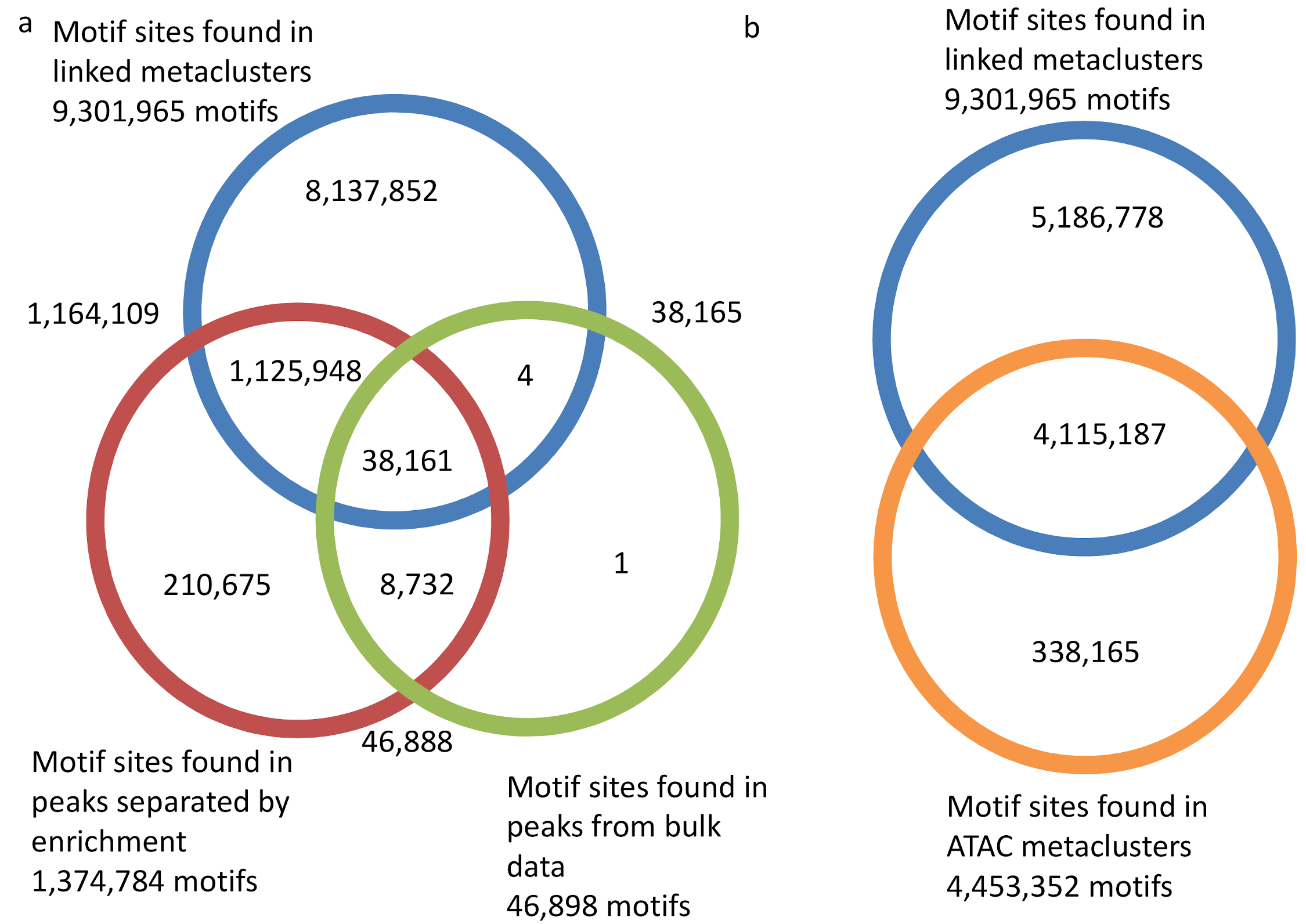

Supplement: S13 Fig — (A) Graph detailing the number of motifs found using the same set of peaks with different groupings using the same q-value < .05 cutoff. (B) Graph detailing the number of motifs found using the same set of peaks with using the linked metacluster grouping and just the ATAC-seq SOM metaclusters grouping using the same q-value < .05 cutoff. (TIF) [file pcbi.1006555.s013.tif]

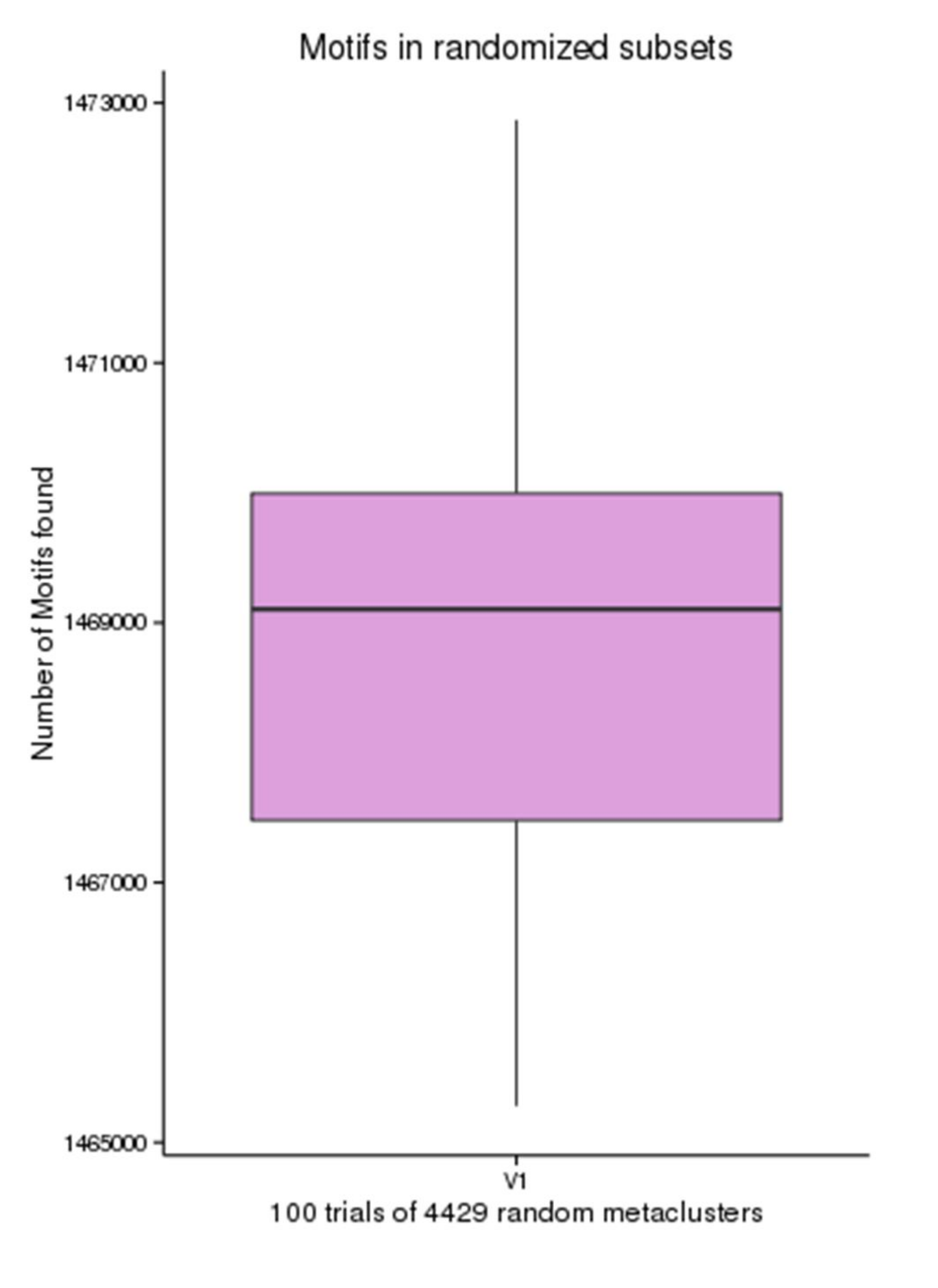

Supplement: S14 Fig — The distribution of motifs found by randomly splitting peaks into 4,429 synthetic linked metaclusters (LM). The mean was ~1,469,000 motifs which is significantly fewer than the ~9.3 million found in the real LMs. (TIF) [file pcbi.1006555.s014.tif]

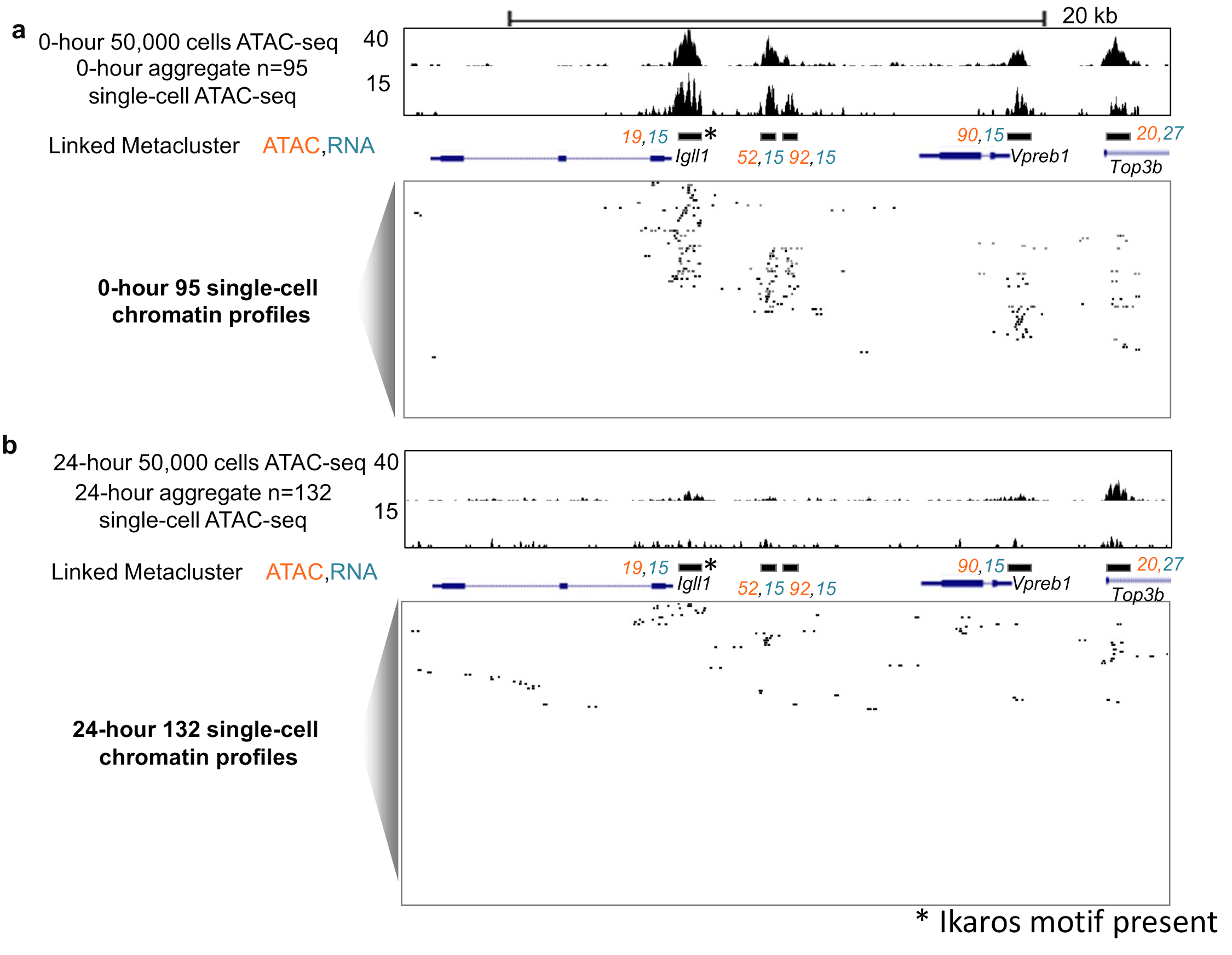

Supplement: S15 Fig — (A-B) UCSC genome browser screenshots of the Igll1 and Vpreb1 loci with bulk (50,000 cells), aggregate (94 single-cells averaged) and single-cell ATAC-seq for 0 (A; 94 single-cells) and 24-hour (B;133 single-cells) pre-B cells. Linked SOM ids (ATAC, RNA) are depicted for all chromatin elements. (TIF) [file pcbi.1006555.s015.tif]

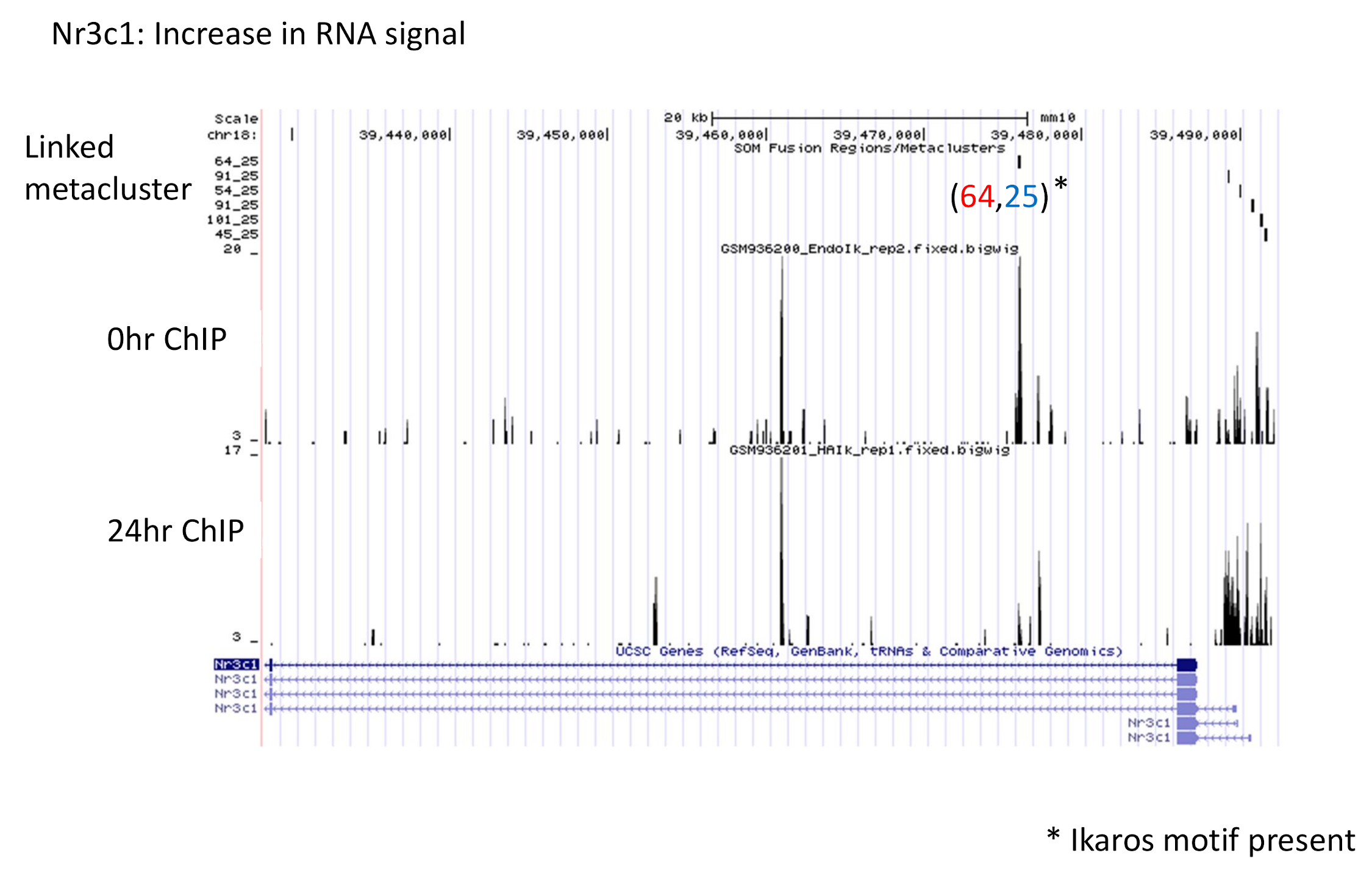

Supplement: S16 Fig — UCSC genome browser snapshots of Ikaros ChIP data taken at the 0-hour and 24-hour timepoints near Nr3c1. The location of the predicted motif is noted along with its linked metacluster ID. The marked location has a significant change in binding at the marked location over the time course. (TIF) [file pcbi.1006555.s016.tif]

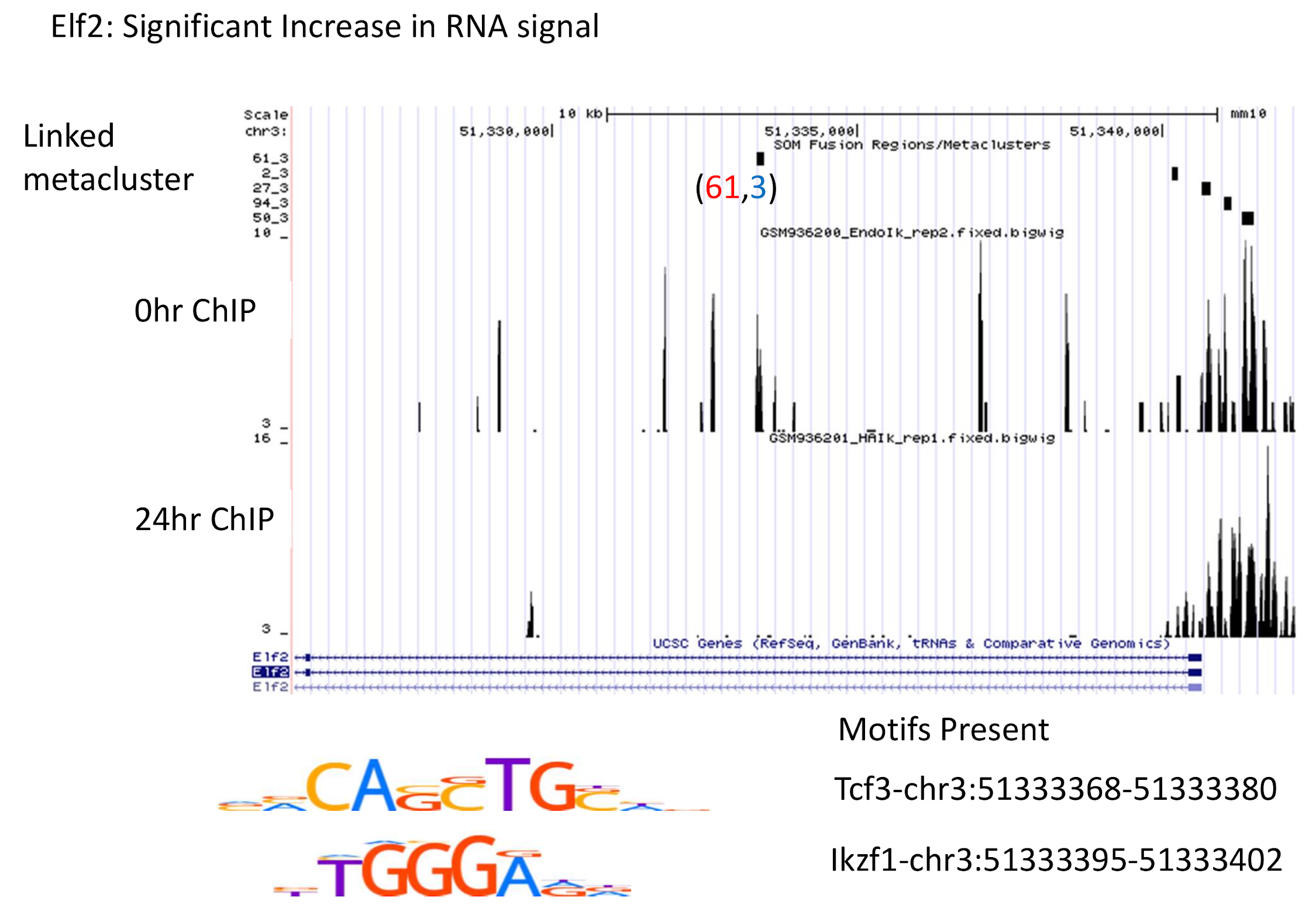

Supplement: S17 Fig — UCSC genome browser snapshots of Ikaros ChIP data taken at the 0-hour and 24-hour timepoints near Elf2. There were 2 predicted motifs in this metacluster, Ikaros and Tcf3. (TIF) [file pcbi.1006555.s017.tif]

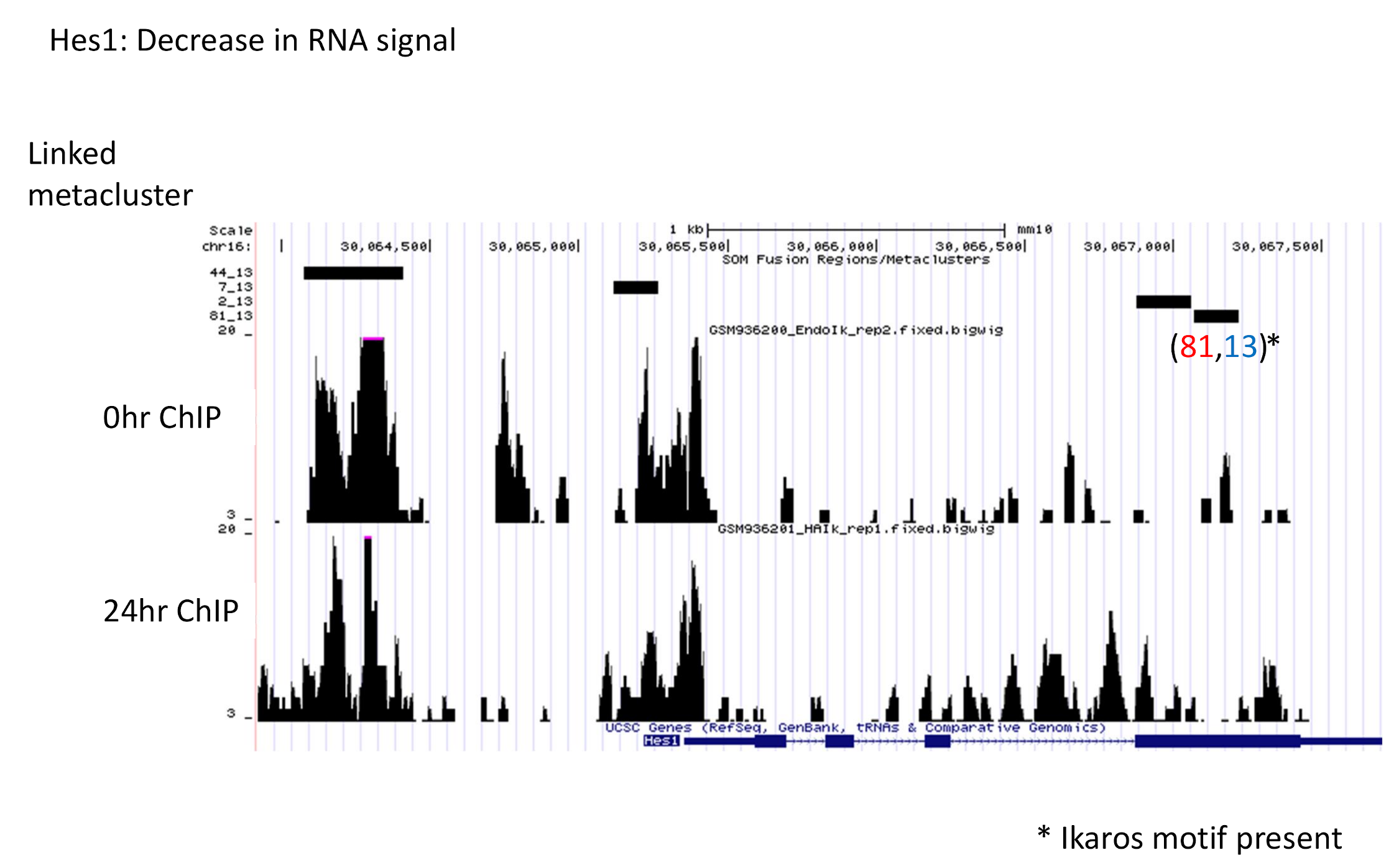

Supplement: S18 Fig — UCSC genome browser snapshots of Ikaros ChIP data taken at the 0-hour and 24-hour timepoints near Hes1. The location of the predicted motif is noted along with its linked metacluster ID. The marked location has a significant change in binding at the marked location over the time course. (TIF) [file pcbi.1006555.s018.tif]

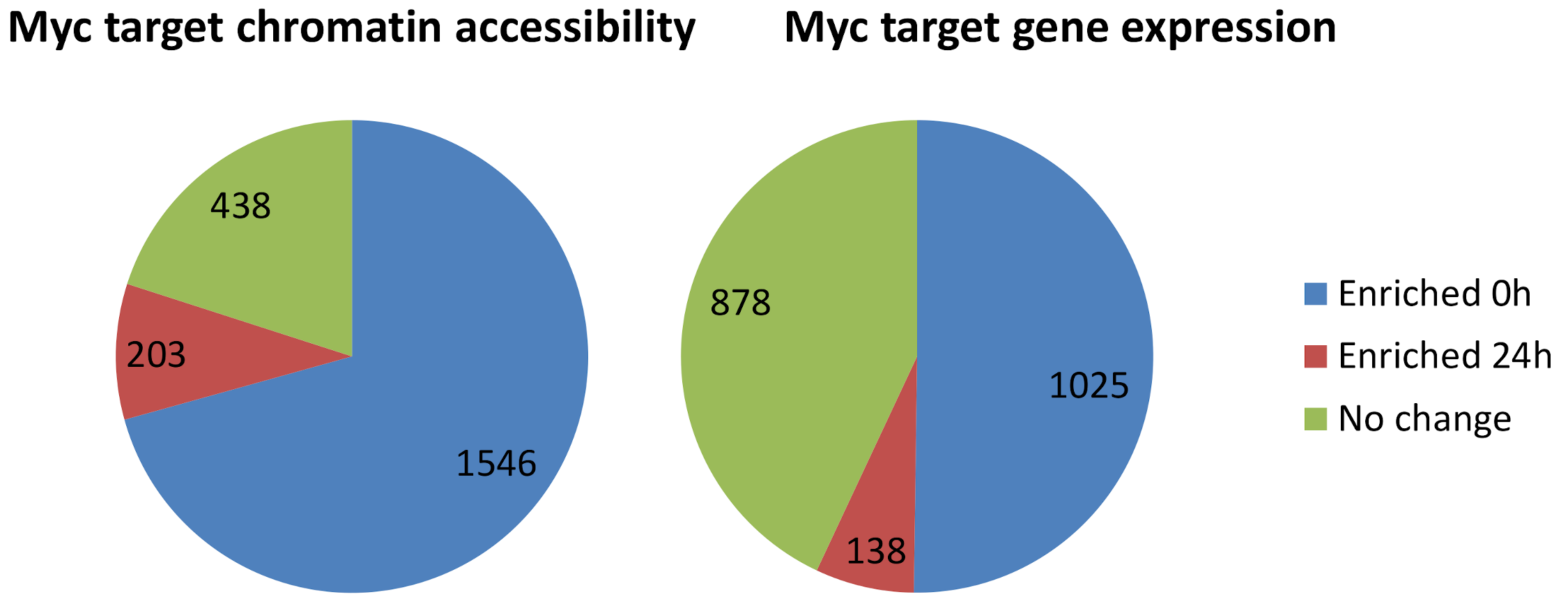

Supplement: S19 Fig — Myc (whose signal drops dramatically from 0- to 24- hour) downstream targets were predicted in a method similar to that in Fig 4. Around half of these react with a drop in signal with a small portion reacting with an increase. This is similar to the change in chromatin accessibility at the predicted binding sites near these genes. (TIF) [file pcbi.1006555.s019.tif]

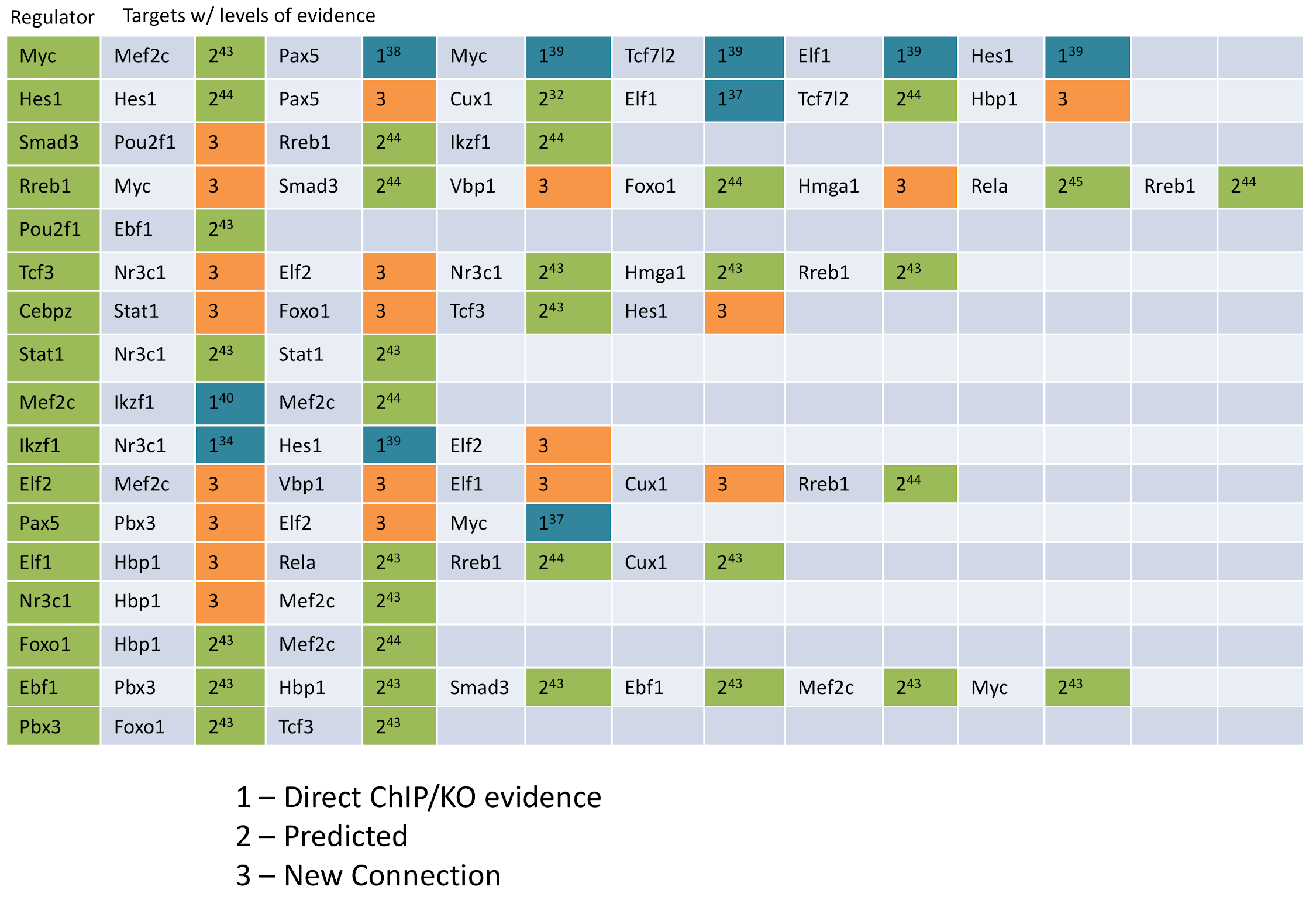

Supplement: S20 Fig — A list of transcription factors with significant changes over the time course and the transcription factors were predicted to regulate. Each regulated gene is followed by a label for the level of existing evidence and reference number if relevant. (TIF) [file pcbi.1006555.s020.tif]

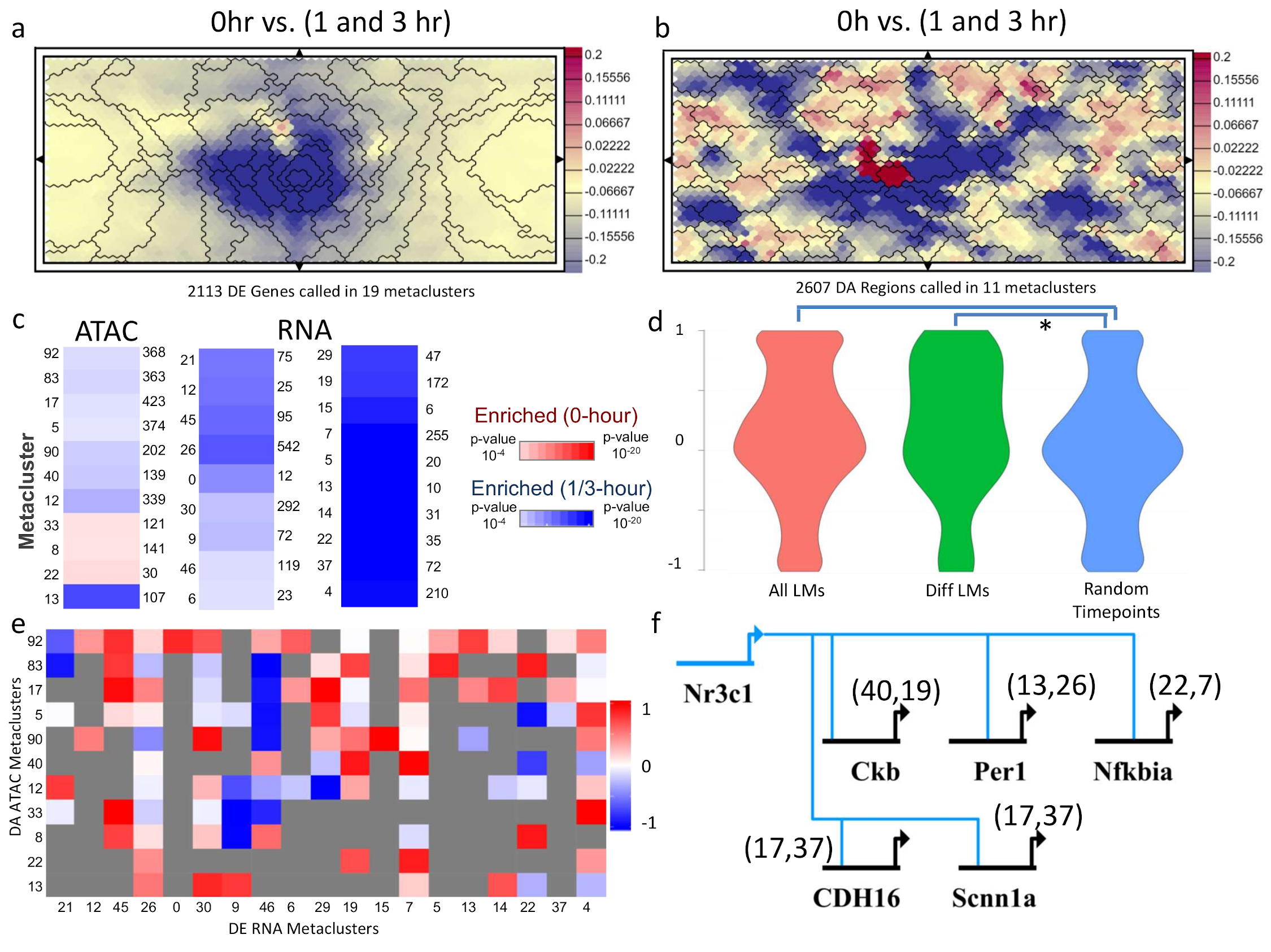

Supplement: S21 Fig — (A-B) Difference maps displaying the areas of temporal enrichment after training on sciCar data. (C) RNA data was differential in 19 metaclusters that represent 2113 genes. The ATAC data was differential in 11 metaclusters representing 2607 genome regions. (D) Violin plots describing the distribution of average temporal correlations between linked ATAC-seq peaks and genes. The differential metaclusters (in green) have fewer combinations with no correlation and more with negative correlations than the distributions from all LMs (in red). Both distributions are significantly (pvalue < .05) different than when the timepoints of the cells are scrambled (in blue). (E) A heatmap of the average temporal correlations from the differential linked metaclusters. (F) Known targets of Nr3c1 (GR receptor) recovered during motif and network analysis. These downstream genes all appeared in differential RNA metaclusters. (TIF) [file pcbi.1006555.s021.tif]

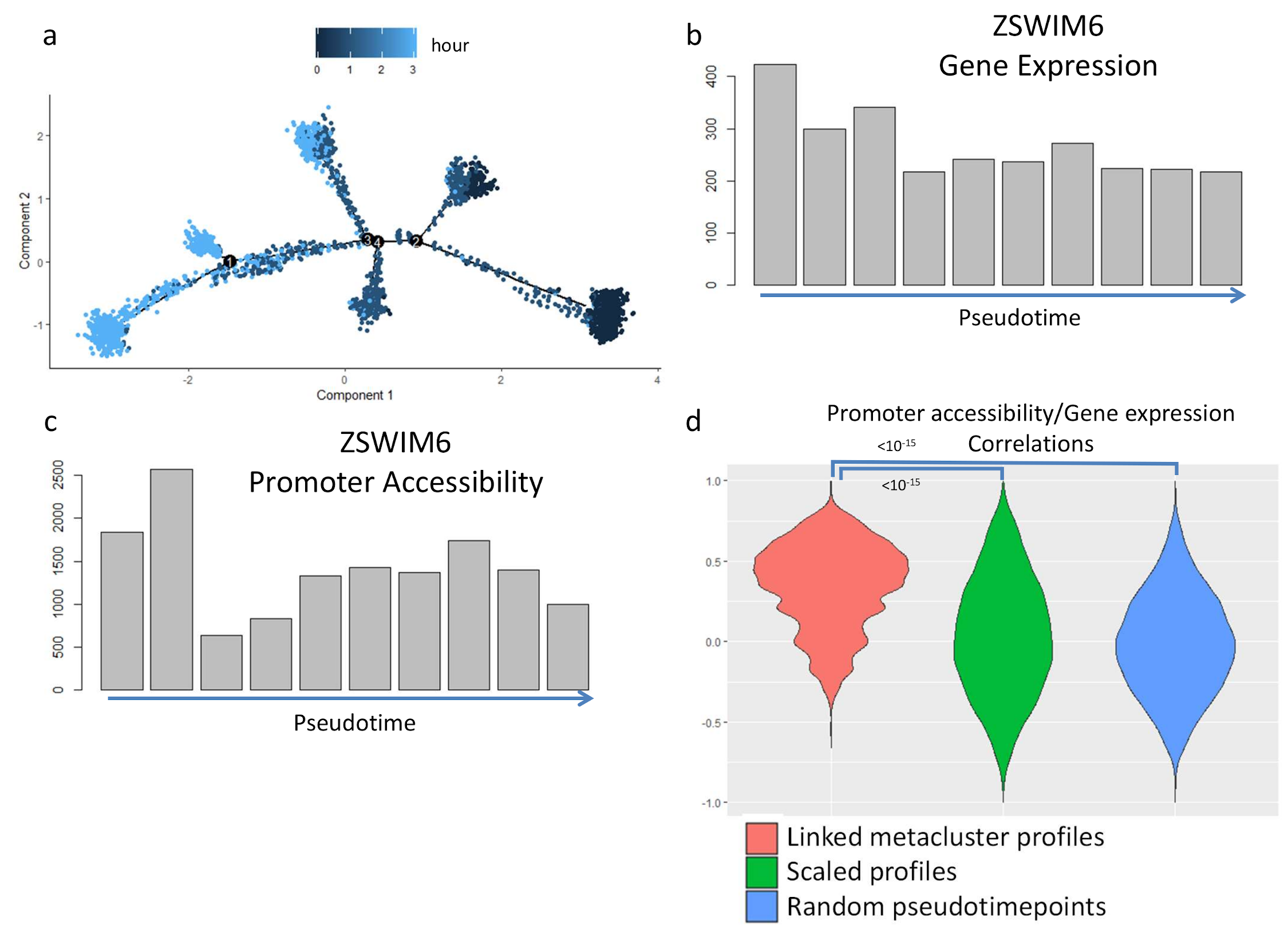

Supplement: S22 Fig — (A) Re-computed pseudotime graph of the scRNA-seq data from the sciCar dataset by monocle [18] is very similar to original publication. (B-C) The scaled signal of the gene expression for ZSWIM6 and chromatin accessibility for its promoter from binned cells is very similar to the original publication. (D) Violin plots of the computed correlations between gene expression and their promoters’ accessibility. Linked metacluster profiles had significantly higher correlations than simply scaling the profiles. (TIF) [file pcbi.1006555.s022.tif]

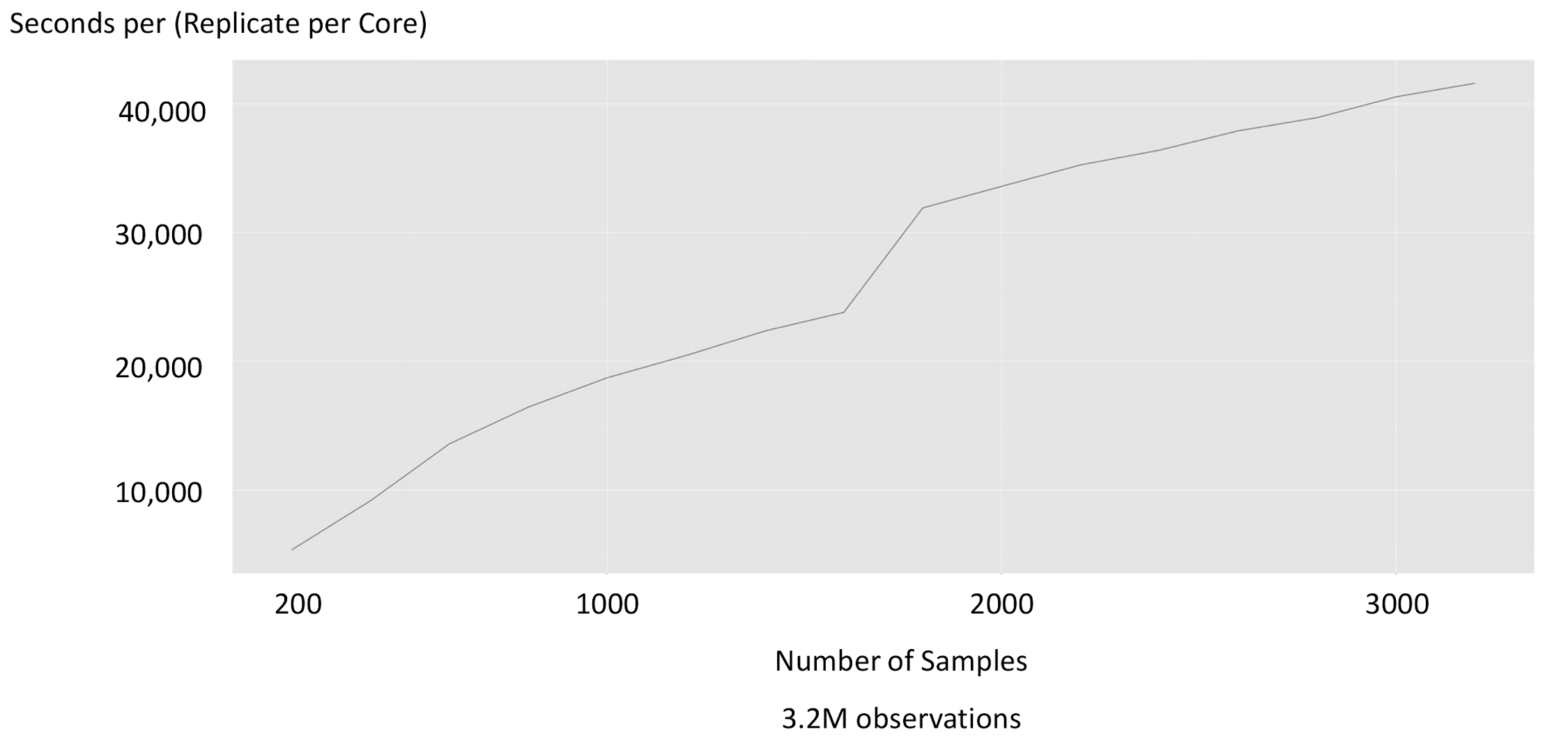

Supplement: S23 Fig — Runtime estimates for SOMatic running on different number of samples. Online SOMs have an inherent complexity of O(Observations*Samples) which SOMatic replicates. (TIF) [file pcbi.1006555.s023.tif]
